# Microphysical Model Predictions of Fault Restrengthening Under Room‐Humidity and Hydrothermal Conditions: From Logarithmic to Power‐Law Healing

**DOI:** 10.1029/2019JB018567

**Published:** 2020-04-03

**Authors:** Jianye Chen, Martijn P. A. van den Ende, André R. Niemeijer

**Affiliations:** ^1^ HPT Laboratory, Department of Earth Sciences Utrecht University Utrecht the Netherlands; ^2^ Faculty of Civil Engineering and Geosciences Technical University of Delft Delft the Netherlands; ^3^ Université Côte d'Azur, CNRS, IRD, Observatoire de la Côte d'Azur, Géoazur Valbonne France

**Keywords:** slide‐hold‐slide experiment, frictional healing, microphysical friction model, non‐Dieterich‐type healing, fault restrengthening, rock friction

## Abstract

The maximum fault strength and rate of interseismic fault strengthening (“healing”) are of great interest to earthquake hazard assessment studies, as they directly relate to event magnitude and recurrence time. Previous laboratory studies have revealed two distinct frictional healing behaviors, referred to as Dieterich‐type and non‐Dieterich‐type healing. These are characterized by, respectively, log‐linear and power‐law increase in the strength change with time. To date, there is no physical explanation for the frictional behavior of fault gouges that unifies these seemingly inconsistent observations. Using a microphysical friction model previously developed for granular fault gouges, we investigate fault strengthening analytically and numerically under boundary conditions corresponding to laboratory slide‐hold‐slide tests. We find that both types of healing can be explained by considering the difference in grain contact creep rheology at short and long time scales. Under hydrothermal conditions favorable for pressure solution creep, healing exhibits a power‐law evolution with hold time, with an exponent of ~1/3, and an “apparent” cutoff time (*α*) of hundreds of seconds. Under room‐humidity conditions, where grain contact deformation exhibits only a weak strain‐rate dependence, the predicted healing also exhibits a power‐law dependence on hold time, but it can be approximated by a log‐linear relation with *α* of a few seconds. We derive analytical expressions for frictional healing parameters (i.e., healing rate, cutoff time, and maximum healing), of which the predictions are consistent with numerical implementation of the model. Finally, we apply the microphysical model to small fault patches on a natural carbonate fault and interpret the restrengthening during seismic cycles.

## Key Points

- We unify two classes of frictional healing behavior with a microphysical model
- We derive analytical expressions for frictional healing parameters (healing rate, cutoff time, and maximum fault strengthening)
- We adopt the physically based model to simulate natural seismic cycle behavior

## 1 Introduction

**Figure 1:**
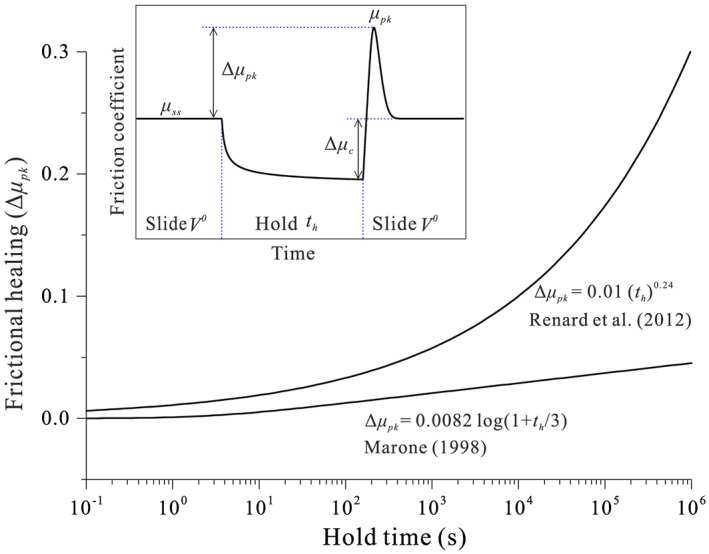
Typical frictional healing behaviors observed in laboratory experiments performed under hydrothermal versus room‐humidity conditions. The inset illustrates the definitions of frictional healing parameters that are commonly obtained from a slide‐hold‐slide test.

For earthquakes to recur on a seismogenic fault, a mechanism is required by which the fault recovers shear strength that was lost during the coseismic phase (Dieterich, 1972). The restrengthening of faults during the interseismic phase (generally referred to as frictional healing) is an important aspect of seismic hazard assessments, as it exerts strong constraints on the recurrence time and magnitude of seismic events. To study fault restrengthening in the laboratory, slide‐hold‐slide (SHS) tests are commonly employed as an analog for seismic cycles (e.g., Marone, 1998; Marone & Saffer, 2015). Laboratory fault restrengthening is often found to increase log linearly with hold time (Dieterich, 1972), which has been termed “Dieterich‐type” healing (DH) and is expressed as *∆μ*_*pk*_ = *β*log(1+*t*_*h*_/*α*), where Δ*μ*_*pk*_ is the increase in peak friction, *t*_*h*_ the hold time, and *β* a constant known as healing rate (typically of the order of 0.01 decade^−1^ or less; Marone, 1998; Carpenter et al., 2016). Further, *α* is the cutoff time of the order of ~1 s, beyond which Δ*μ*_*pk*_ conforms to a log‐linear relation with *t*_*h*_ (Figure 1). As observed in SHS tests, this parameter can be sometimes interpreted as the effective contact time at the beginning of a hold period, which is inversely proportional to the prehold sliding velocity (Marone, 1998). Such frictional healing is one key manifestation of the “evolution effect” in classical rate‐ and state‐dependent friction (RSF) laws (Dieterich, 1979; Ruina, 1983), which has been argued to relate to the evolution of the real contact area of load‐bearing interface asperities. Using in situ microscopic imaging techniques, the logarithmic growth of contact area with time has indeed been observed on the frictional interfaces of PMMA, halite, quartz, and calcite (e.g., Dieterich & Kilgore, 1994, 1996; Renard et al., 2012), even though the underlying mechanisms are yet to be unambiguously identified.

Recently, more and more exceptions to these general results have been reported for gouge‐filled laboratory faults, which can generally be referred to as “non‐Dieterich‐type” healing (NDH). Many of these exceptions share a common interpretation that NDH is promoted under hydrothermal conditions where fluid‐assisted mass transfer processes, such as pressure solution, are efficient (e.g., Nakatani & Scholz, 2004). Under such conditions, frictional healing is typically characterized by a (stronger than logarithmic) power‐law growth of static friction with hold time (Bos & Spiers, 2002; Yasuhara et al., 2005), exhibiting a healing rate *β* and cutoff time *α* that are much larger than observed under room‐humidity conditions (e.g., *β*~0.24, Nakatani & Scholz, 2004; *α*~1,000 s, Niemeijer et al., 2008). The substantial differences in the rate and type of frictional healing observed in laboratory experiments pose major challenges for the extrapolation of laboratory results to natural scales and conditions (e.g., Carpenter et al., 2014), which in turn negatively impacts the confidence with which to interpret seismic hazard assessments.

Despite considerable efforts made to elucidate the mechanics of frictional healing through laboratory experiments from which a number of physical mechanisms have previously been identified (e.g., contact growth and cementation), up to now, there is no quantitative microphysical model that can unify the different observations. One common interpretation is that the time dependence of rock peak friction originates from an increase in real contact area as a result of a thermally activated creep process operating on the interface contact asperities (e.g., Dieterich & Kilgore, 1994; Estrin & Bréchet, 1996; Nakatani & Scholz, 2006) and/or from an increase in intrinsic contact junction strength (e.g., Li et al., 2011). However, it has long been recognized that the classical RSF laws cannot readily explain the diverse frictional healing behaviors observed in laboratory studies (e.g., Beeler et al., 1994; Bhattacharya et al., 2017; Chester & Higgs, 1992). In part, this may be due to the fact that original physical explanations focus on bare rock surfaces based on a conceptual model of contact asperity growth and annihilation. These models do not consider the dilatation/compaction processes that are widely observed to operate in fault gouges (e.g., Beeler & Tullis, 1997; Niemeijer & Spiers, 2006; Richardson & Marone, 1999). Without a microphysical basis to account for such effects (Segall & Rice, 1995; Sleep, 1995; Sleep et al., 2000), to extrapolate the laboratory healing data to natural fault systems, notably beyond laboratory time scales, involves significant and often unknown uncertainties (e.g., Carpenter et al., 2014; Ikari et al., 2016; Marone & Saffer, 2015).

By considering the microscale physics operating in a shearing fault gouge, Chen and Spiers (2016) have previously developed a microphysical model elaborating on the work of Niemeijer and Spiers (2007) (hereafter referred to as the Chen‐Niemeijer‐Spiers or “CNS” model). Using independently measured parameter values, the CNS model is able to simulate a range of laboratory friction tests, including constant‐velocity, velocity‐stepping, and SHS tests, and produce results that capture the main features and trends seen in the experiments, with reasonable quantitative agreement (Chen et al., 2017; Hunfeld et al., 2019). Note that the present model is not derived to account for clay‐rich fault gouges (i.e., with a matrix‐supported structure), which may require a more microstructurally complex model such as the one presented by den Hartog and Spiers (2014). In the present study, we investigate fault restrengthening in the framework of the CNS model, aiming to help resolve the long‐existing debate of logarithmic versus power‐law frictional healing from a microphysical basis, that is, from the perspectives of operating rock deformation mechanism(s) under in situ, temperature‐pressure‐fluid conditions. The governing equations are solved analytically under boundary conditions corresponding to typical SHS tests, from which physical expressions for frictional healing parameters are obtained. The resulting model predicts distinct healing behaviors for different conditions, showing a broad agreement with previous laboratory observations. Encouraged by these results, we subsequently discuss the implications for interseismic healing of fault systems in nature.

## 2 The Microphysical Model (CNS Model)

**Figure 2:**
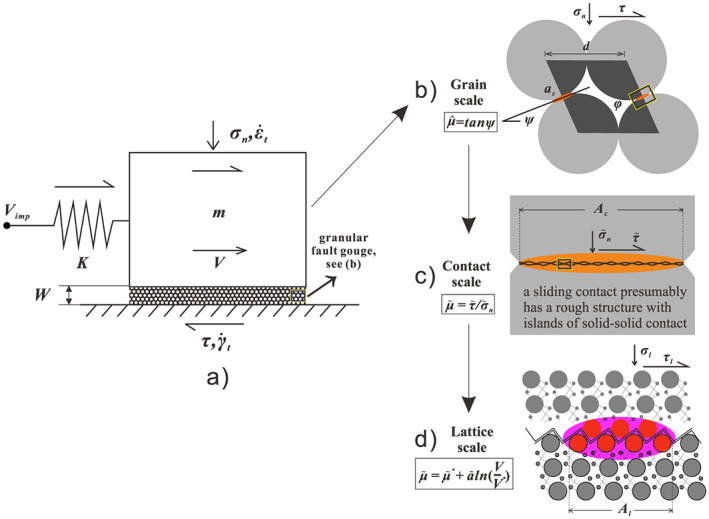
(a) Schematic diagram of a single degree of freedom elastic system representing a fault zone system. A slider moves along a frictional interface at a possibly varying slip rate *V*, which is driven by an imposed velocity *V*_imp_, with stress transmitted through a spring with stiffness *K*. The mass of the slider is neglected. The friction interface is assumed to be filled with granular fault gouge that has an idealized (steady state) microstructure, as shown in Figures 1b–1d. (b) The unit cell characterizing the grain pack in the microstructural model, in which the key microstructural state variables, that is, porosity (*φ*), average contact area (*a*_*c*_), and dilatancy angle (*ψ*) are illustrated, corresponding to a frictional strength due to intergranular dilatation ( $\hat{\mu }\equiv \tan\psi$). (c) Illustration of a single contact at the contact scale, which has an “apparent” overlapping area *A*_*c*_ but is essentially characterized by a rough structure with islands of solid‐solid contact (asperities). (d) Illustration of a single asperity at the lattice scale, which has a solid‐to‐solid junction area *A*_*l*_ and shows rate strengthening of grain boundary friction (Chen & Spiers, 2016). Across these different scales, the externally applied effective normal and resulting shear stresses (*σ*_*n*_ and *τ*) are transmitted across the grain contacts, causing intensified local stresses on the contacts ( ${\tilde{\sigma }}_{n}$ and $\tilde{\tau }$) and asperities (*σ*_*l*_ and *τ*_*l*_).

A detailed derivation of the CNS model framework is presented by Niemeijer and Spiers (2007) and Chen and Spiers (2016). For convenience, here we only summarize some of the key aspects of this model. The model is generally applicable to both laboratory and natural faults filled with granular gouge material (Figure 2). In the CNS model, the microstructure of a granular gouge is ideally represented by an arrangement of densely packed spheres. Presently, we only consider the frictional phenomenon after the material has reached a well‐matured microstructure and thus the model does not include changes in grain size and neglects mechanisms such as cataclasis and grain comminution, as well as progressive (de)localization. The microstructural state and the overall frictional strength of the gouge are controlled by two competing processes: dilatation due to granular flow and time‐dependent compaction. As derived from thermodynamic and kinematic considerations, the governing equations are written ass

(1a) $$\frac{\dot{\tau }}{K}=V_{\mathrm{imp}}-W\left({\dot{\gamma }}_{\mathrm{pl}}+{\dot{\gamma }}_{\mathrm{gr}}\right),$$

(1b) $$\frac{\dot{\varphi }}{\left(1-\varphi \right)}={\dot{\gamma }}_{\mathrm{gr}}\tan\psi -{\dot{\varepsilon }}_{\mathrm{pl}},$$

(1c) $$\tau =\frac{\tilde{\mu }+\tan\psi }{1-\tilde{\mu }\tan\psi }\sigma_{n},$$

(1d) $$\tilde{\mu }={\tilde{\mu }}^{*}+a_{\tilde{\mu }}\ln\left({\dot{\gamma }}_{\mathrm{gr}}/{\dot{\gamma }}_{\mathrm{gr}}^{*}\right).$$

These relations can be conceptually understood as follows:

1. Equation 1a expresses the fault‐parallel kinematic relation, and we represent the fault as a one‐dimensional, spring‐block system composed of a linear spring activated by the imposition of a velocity at a load point, assuming no inertia (Figure 2a). In this equation, *τ* is shear stress; *V*_imp_, imposed displacement rate; *K*, spring stiffness; and *W*, shear zone thickness. The sample deformation results from the parallel operation of “plastic” creep ${\dot{\gamma }}_{\mathrm{pl}}\;$ and granular flow ${\dot{\gamma }}_{\mathrm{gr}}$ in the shear direction. For the purpose of this study, deformation is assumed to be uniform over the shear zone (Figure 2a). In the case that slip is localized, we neglect the deformation in the bulk layer, and *W* is set the thickness of the shear band.
2. Equation 1b expresses the deformation in the fault‐normal direction (i.e., volumetric strains), where *φ* is the porosity of the shear zone and ${\dot{\varepsilon }}_{\mathrm{pl}}\;$ is the normal strain rate associated with creep‐induced compaction. Granular flow induces dilatation, which can be expressed as $\;{\dot{\varepsilon }}_{\mathrm{gr}}=-{\dot{\gamma }}_{\mathrm{gr}}\tan\psi$, where *ψ* is the average dilatancy angle (Figure 2b).
3. Equation 1c is the “friction law” of the CNS model for the friction regime (i.e., when granular flow dominates over plastic creep), given that shear strength can be expressed in terms of two components, namely, grain boundary (gb) friction $\;\tilde{\mu }\;$ and friction due to intergranular dilatation $\;\hat{\mu }\equiv \tan\psi$ (see the definitions in Figures 2c and 2b, respectively).
4. We adopt a cohesion‐free gb slip criterion (equation 1d). As derived from the lattice scale, the gb slip is intrinsically rate strengthening in a logarithmic form, where $a_{\tilde{\mu }}\;$ is a strain rate‐dependent coefficient and ${\tilde{\mu }}^{*}\;$ is the gb friction coefficient at a reference strain rate ${\dot{\gamma }}_{\mathrm{gr}}^{*}$ (Figure 2d).

In the CNS model, porosity (*φ*), average dilatancy angle (*ψ*), and average contact area (*a*_*c*_) form a set of three, geometrically interrelated state variables characterizing the microstructural state of a deforming fault gouge and given by the approximate relations (Niemeijer & Spiers, 2007; Spiers et al., 2004)

(2a) $$\tan\psi =H\left(q-2\varphi \right),\text{and}$$

(2b) $$a_{c}=\pi d^{2}\left(q-2\varphi \right)/z,$$

where *H* is a constant related to the packing geometry of the grains, *q* is a geometric constant with a value of two times the critical‐state porosity (*q* = 2*φ*_*c*_), *d* is nominal grain diameter (Figure 2b), and *z* is the average coordination number. Since these three variables are dependent on one another through the microstructure, the CNS model can be idealized as a single‐state‐variable system. Equation 1b is also referred to as the “state equation,” as it is a natural choice to express the state in terms of a microstructurally observable quantity (porosity). Since $\tilde{\mu }$ is strain rate dependent and $\hat{\mu }$ is a result of the volumetric (compaction) “state” of the gouge, our friction law (equation 1c) also expresses the frictional strength as a function of “rate” and “state.”

The constitutive equation set (1a)–(1d) can be written into a pair of ordinary differential equations, specifying the rate of change of shear stress (*τ*) and state (*φ*). Following previous work (Chen & Spiers, 2016), the two ordinary equations are solved using the finite element package COMSOL. To avoid unstable sliding and to get a clear peak healing that allows for comparison with the analytical results, we use a high spring stiffness (6.0 × 10^11^ Pa/m, 10 times the apparatus stiffness employed by Chen et al., 2015).

## 3 Prediction of Frictional Healing

### 3.1 Characteristic SHS Behavior in Spring‐Block Simulations

**Figure 3:**
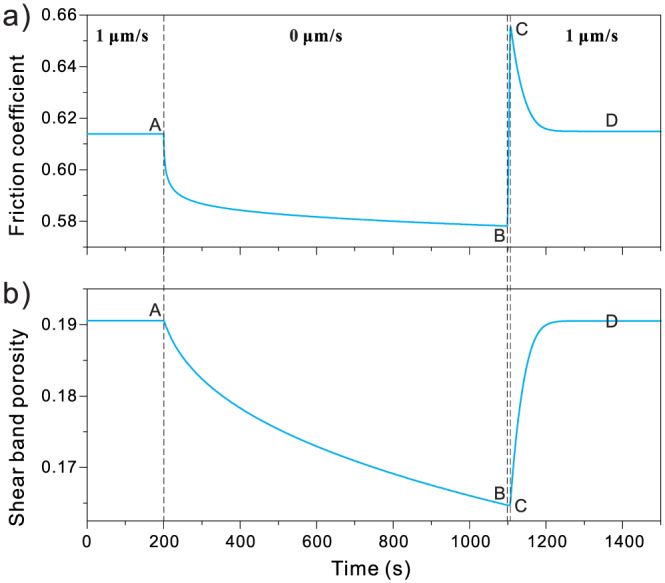
Evolution of (a) friction coefficient and (b) porosity of (localized) shear band during a typical SHS test, as predicted by the CNS model for a carbonate gouge sheared under hydrothermal conditions (*T* = 80 °C and *σ*_*n*_ = 50 MPa, as in Chen & Spiers, 2016).

To gain some initial insight into the model behavior, we first simulate an SHS sequence using a spring‐block analog. We adopt the model settings of Chen and Spiers (2016), which consider a 50‐μm thick shear band localized at the margin of a 0.8‐mm thick calcite gouge layer, with nominal grain sizes of 2 and 20 μm in the shear band and bulk gouge, respectively. Figure 3 gives the prediction of an SHS sequence at velocity (*V*) of 1 μm/s under hydrothermal conditions (*T* = 80 °C and *σ*_*n*_ =50 MPa). As anticipated, the model displays stress relaxation during hold periods (A → B), followed by a rapid increase in shear stress upon reloading (B → C), and a subsequent gradual decay to steady state (C → D; Figure 3a). The gouge compacts during the hold period, and upon reshearing it, begin to dilate to gradually retain the steady‐state value of porosity (Figure 3b). All these results generally agree with the shear strength evolution and fault‐normal displacement observed in laboratory experiments (e.g., Beeler & Tullis, 1997; Dieterich, 1979). Neglecting deformation in the bulk layer generates the same results, including the evolution of shear stress and porosity, with the exception that less total compaction is produced. Since the bulk gouge does not exhibit granular flow, compaction of the bulk gouge does not affect the shear strength of the sample.

**Figure 4:**
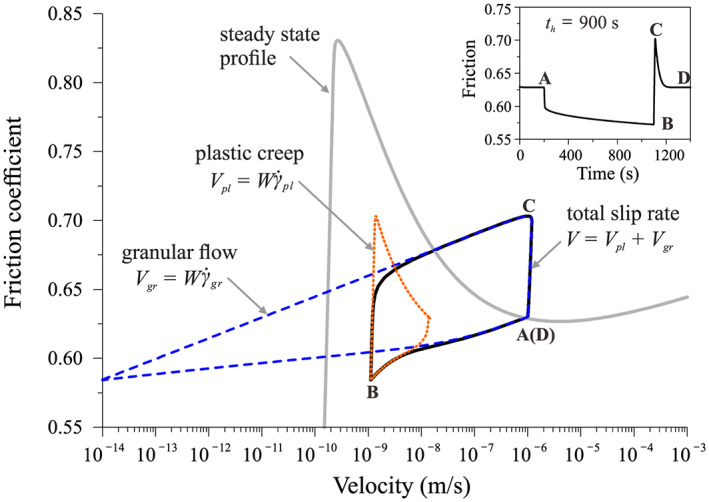
Friction‐velocity phase diagram for the SHS sequence shown in Figure 3, with a hold time of 900 s (in solid black line). The steady‐state profile showing the steady‐state friction coefficient as a function of log (velocity) is added for comparison (in a thick gray line). The red and blue dashed lines illustrate that the sample deformation rate (*V*) consists of two components, namely, granular slip rate ( $V_{\mathrm{gr}}=W{\dot{\gamma }}_{\mathrm{gr}}$) and shear creep rate ( $V_{\mathrm{pl}}=W{\dot{\gamma }}_{\mathrm{pl}}$), where *W* is the shear zone thickness. The marked points A, B, C, and D are the same as those in Figure 3.

To more clearly elucidate the deformation mechanism(s) during the SHS sequence, we plot the result in the friction coefficient‐velocity phase diagram (Figure 4). Here, the sample velocity (*V*) consists of two components, namely, *V*_gr_ = *W* ${\dot{\gamma }}_{\mathrm{gr}}$ and *V*_pl_ = *W* ${\dot{\gamma }}_{\mathrm{pl}}$, by granular flow and “plastic” creep, respectively (see their time evolution in Supporting Information Figure S2). During stable sliding (A), the rate of granular flow is orders of magnitude faster than that of shear creep on the grain contacts, accommodating most of the imposed displacement. During the hold, the shear deformation persists by elastic unloading of the system, through which the shear stress relaxes and the strain rate decreases with time (A → B). During early stages of the hold (i.e., <200 s; Figure S2), the slip is mostly accommodated by granular flow, which, with the passage of time, decreases dramatically so that shear creep becomes dominant. Upon resliding, the shear strain rate recovers in a very short time (B → C), and the peak strength is achieved when the sample velocity *V* reaches the imposed velocity *V*_imp_ (C).

### 3.2 Frictional Healing Under Hydrothermal Conditions

In the following, we present an analytical solution for the CNS model in response to an SHS sequence. We first take the hydrothermal case, where diffusion‐controlled intergranular pressure solution (IPS) is assumed to operate at the grain contacts, following a linearized creep law (Spiers et al., 2004).

(3) $${\dot{\varepsilon }}_{\mathrm{pl}}=A_{d}\frac{\mathrm{DCS}}{d^{3}}\frac{\sigma_{n}\Omega }{\mathrm{RT}}f\left(\varphi \right).$$

Here, *σ*_*n*_ is the applied effective normal stress, *f*(*φ*) is a dimensionless function of porosity and all the other parameters are considered to be constant (*A*_*d*_, a geometric constant; *C*, solubility of the solid phase in m^3^/m^3^; *S*, thickness of the gb fluid phase in m; *D*, diffusivity of the solute within the fluid in m^2^/s; Ω, molar volume of the solid phase in m^3^/mol; *R*, gas constant at 8.31 Jmol^−1^ K^−1^); *T*, absolute temperature in K; and *d* is the average grain size). In general, the porosity function can be written as

(4) $$f\left(\varphi \right)={\left(q-2\varphi \right)}^{-p},$$

where *p* is a sensitivity factor that accounts for the changes of the contact area (*a*_*c*_, hence contact stress magnitude) and the length of the diffusion path (if diffusive mass transfer is limiting the overall rate of pressure solution), with changing porosity. For the diffusion‐controlled IPS, *p* assumes a value of 2, and for interface reaction‐controlled IPS, *p* assumes a value of 1 (Spiers et al., 2004).

#### 3.2.1 Gouge Compaction During a Hold Period

Based on the above creep laws, the time‐dependent compaction during a hold period (*t*) can be derived from equation 1b as follows. Without loss of generality, we assume the prehold, stable sliding rate as a reference ( ${\dot{\gamma }}_{\mathrm{gr}}^{*}={\dot{\gamma }}_{\mathrm{gr}}^{\mathrm{ss}}$). At steady state, the time derivative of porosity is 0, giving the relation ${\dot{\varepsilon }}_{\mathrm{pl}}^{\mathrm{ss}}=\tan\psi_{\mathrm{ss}}{\dot{\gamma }}_{\mathrm{gr}}^{*}$. At the moment the load point is arrested, the gouge will compact following equation 1b. Incorporating equations 2a, 2b, 3, 4a, and 4b, equation 1b becomes

(5) $$\frac{d\left(\tan\psi \right)}{\mathrm{dt}}=2H\left(1-\varphi \right){\dot{\gamma }}_{\mathrm{gr}}^{*}\left[\frac{\tan^{3}\psi_{\mathrm{ss}}}{\tan^{2}\psi }-\tan\psi \frac{{\dot{\gamma }}_{\mathrm{gr}}}{{\dot{\gamma }}_{\mathrm{gr}}^{*}}\right].$$

For mathematical convenience, we define $\Psi \equiv {\left(\frac{\tan\psi }{\tan\psi_{\mathrm{ss}}}\right)}^{3}$, which renders equation 5 into

(6) $$\dot{\Psi }=\frac{1}{t_{c}}\left(1-\Psi \frac{{\dot{\gamma }}_{\mathrm{gr}}}{{\dot{\gamma }}_{\mathrm{gr}}^{*}}\right).$$

Here, *t*_*c*_ is the characteristic time *t*_*c*_ = *D*_*c*_/*V*_imp_, where *D*_*c*_ is the characteristic slip distance as embodied in the CNS model: $D_{c}=\frac{W}{6H\left(1-\varphi_{\mathrm{ss}}\right)}$, where *W* is shear zone thickness and *φ*_*ss*_ is the steady‐state porosity at the prehold sliding velocity (hereafter subscript “ss” is reserved for “steady state”). Note that in the CNS model, equivalent rate‐and‐state friction parameters (in the limit of steady state), *a*, *b*, and *D*_*c*_, can be explicitly expressed with fault creep and microstructural parameters (Table S1; Chen et al., 2017). Interestingly, equation 6 shares the same form as the slowness law when using the dimensionless state (Θ = *θ*/θ^*^, where θ^*^ = *D*_*c*_/*V*_imp_ is the reference state in the RSF framework).

The granular slip rate decreases rapidly during the hold time so that for $\Psi \frac{{\dot{\gamma }}_{\mathrm{gr}}}{{\dot{\gamma }}_{\mathrm{gr}}^{*}}\ll 1$, equation 6 reduces to $\;\dot{\Psi }=1/t_{c}$. Applying this asymptote, equation 6 yields the following solution:

(7) $$\tan\psi \left(t\right)=\tan\psi_{\mathrm{ss}}{\left(1+\frac{t}{\alpha }\right)}^{1/3}.$$

Here, *ψ*_ss_ is the steady‐state dilatation angle before hold, and *α* = *t*_*c*_ is the cutoff time. This gives the asymptotic state evolution for long hold times that satisfy the relation $\Psi \frac{{\dot{\gamma }}_{\mathrm{gr}}}{{\dot{\gamma }}_{\mathrm{gr}}^{*}}\ll 1$.

For the initial stage of a hold period, the relaxed shear stress is described by the relation $\;∆\tau_{c}=-a\sigma_{n}\ln\left(1+\frac{t}{t_{\text{in}}}\right)$, where $\;t_{\text{in}}=\frac{a\sigma_{n}}{KV_{\mathrm{imp}}}$ is the characteristic instantaneous response time and *a* is the equivalent parameter of the direct effect as embodied in the CNS model: $a=\frac{\partial \mu }{\partial \ln V}=a_{\tilde{\mu }}\frac{1+{{\tilde{\mu }}_{\mathrm{ss}}}^{2}}{{\left(1-{\tilde{\mu }}_{\mathrm{ss}}\tan\psi_{\mathrm{ss}}\right)}^{2}}$ (Table S1). This is intuitive since the relaxation for the early stages of the hold should mimic the direct response to a velocity downstep. Combining this relation (equation 7) with equation 1a gives the slip rate during the “direct” response, that is, $\frac{{\dot{\gamma }}_{\mathrm{gr}}}{{\dot{\gamma }}_{\mathrm{gr}}^{*}}=1+\frac{t}{t_{\text{in}}}$ ( $V\approx W{\dot{\gamma }}_{\mathrm{gr}}$). The state evolution for the initial stage of the hold can be described as

(8a) $$\dot{\Psi }=\frac{1}{t_{c}}\left(1-\frac{\Psi }{1+t/t_{\text{in}}}\right).$$

To facilitate integration, hold time is normalized as t_*_ = *t*/*t*_*in*_, reducing equation 8a to

(8b) $$\frac{d\Psi }{{\mathrm{dt}}_{*}}=r\left(1-\frac{\Psi }{1+t_{*}}\right)\;\;\text{and}\;r=\frac{t_{\text{in}}}{t_{c}}=\frac{a\sigma_{n}}{{\mathrm{KD}}_{c}}.$$

Satisfying Ψ(0) = 1 for the steady state just before hold, the equation (Bowden & Tabor, 1964) has the solution

(9a) $$\Psi \left(t_{*}\right)=\frac{1+r{\left(t_{*}+1\right)}^{\left(r+1\right)}}{\left(1+r\right){\left(t_{*}+1\right)}^{r}}.$$

Without great sacrifice of accuracy (see the justification in Text S1 and Figure S1), this solution can be reshaped into

(9b) $$\Psi \left(t_{*}\right)\approx 1+\frac{r}{\left(r+1\right)}t_{*}.$$

Back substitution for Ψ and t_*_ yields the state evolution expressed as

(10) $$\tan\psi \left(t\right)=\tan\psi_{\mathrm{ss}}{\left(1+\frac{t}{\alpha }\right)}^{1/3}.$$

In contrast to equation 7, the cutoff time here is defined slightly differently (*α* = *t*_*c*_+*t*_*in*_).

Taken together, the state evolution can be described by the general functional form of equation 7 or 10 for both early and late stages of a hold. For long time scales (*t* ≫ *t*_*c*_), *α* asymptotically approaches *t*_*c*_. For short hold times (less than a few times of *t*_*c*_), a closer solution is obtained with *α* = *t*_*c*_+*t*_*in*_, where $\;t_{\text{in}}=\frac{a\sigma_{n}}{KV_{\mathrm{imp}}}$. Using parameters from typical calcite friction experiments (*D*_*c*_ = 20 μm, *V*_imp_ = 1 μm/s, *a* = 0.006, and *K*/*σ*_*n*_ = 0.001 μm^−1^) gives *t*_*c*_ of 20 s and *t*_*in*_ of 6 s. In principle, it is the dominant deformation mechanism that distinguishes between the short and long hold times (short times by intergranular slip and long ones by shear creep at grain contacts), which can be determined from the numerical results (Figures 4 and S2).

#### 3.2.2 Fault Restrengthening Upon Reshear

Upon reshear from a hold, shear stress will first follow the elastic loading curve. At the peak stress, the derivative of shear stress in equation 1a is zero so that *V* must be equal to $V_{\mathrm{imp}}\;\left(\approx W{\dot{\gamma }}_{\mathrm{gr}}^{*}\right)$, leading to $\tilde{\mu }={\tilde{\mu }}^{*}$. The peak friction can be hence approximated as $\mu_{\mathrm{pk}}=\frac{{\tilde{\mu }}^{*}+\tan\psi }{1-{\tilde{\mu }}^{*}\tan\psi }$. By definition, frictional healing is calculated as *∆μ*_*pk*_ = *μ*_*pk*_ − *μ*_ss_. Using the equivalent *b* value (Table S1) and equation 7, we obtain

(11) $$∆\mu_{\mathrm{pk}}\left(t\right)=3b\left[\frac{\tan\psi \left(t\right)}{\tan\psi_{\mathrm{ss}}}-1\right]=3b\left[{\left(1+\frac{t}{\alpha }\right)}^{1/3}-1\right].$$

This equation demonstrates that frictional healing is governed by the state change achieved over the hold period and follows a power‐law relation in time. Analogous to the RSF framework, healing rate can be determined as *β* = d(*∆μ*_*pk*_)/d(log*t*). In the case that *t*_*h*_ ≫ *α*, the relation leads to

(12) $$\beta =2.3b{\left(1+\frac{t}{\alpha }\right)}^{1/3}.$$

This relation shows that the healing rate as defined in RSF framework is not a constant but rather is a power‐law function of hold time.

### 3.3 Frictional Healing Under Room‐Humidity Conditions

Under room‐humidity conditions (for which fluid‐rock interactions are often slow or absent), the dominant deformation mechanisms for “creep” on grain contacts (or interiors) are ambiguous: subcritical cracking, twinning and kinking, and dislocation cross slip and dislocation glide are all candidate mechanisms. In the absence of tight constraints on the exact deformation mechanism, we here adopt a general creep law in a power form (Chen et al., 2017):

(13) $$\dot{\varepsilon }\equiv E_{\mathrm{pl}}\left(T,d,\sigma_{n}\right)=B\left(T,d\right){\sigma_{n}}^{n}.$$

In this equation, *σ*_*n*_ is the applied effective normal stress, *n* is the stress exponent, and *B*(*d*, *T*) is a kinetic constant (in units of Pa^1/*n*^ s^−1^), constituting the temperature (*T*) and grain size (*d*) dependencies of the creep law. Correspondingly, the strain‐rate sensitivity of stress ( $d\left(\log\sigma_{n}\right)/d\left(\log\dot{\varepsilon }\right)$) grows as 1/*n* so that a large stress exponent results in low sensitivity. Typically, subcritical cracking has a stress exponent of 10–30 (for ceramics; Munz & Fett, 1999). Dislocation creep has a theoretical *n* value of 3–5, although higher values are commonly observed, especially at low temperatures (5–12, by cross slip for calcite; De Bresser, 2002). Twinning and kinking also show a weak sensitivity to the applied shear stress, with a typical large *n* value (e.g., >10 for calcite; Rybacki et al., 2013). Owing to the extremely weak stress dependence, dislocation glide is often described by an exponential relation. If the data were fitted with a power law, even larger *n* values would be obtained (*n*~67 for muscovite; e.g., Mares & Kronenberg, 1993). In the case that one of the aforementioned mechanisms governs “creep” of a “dry” gouge, a much weaker strain‐rate dependence (*n* >> 1) is anticipated than for creep at hydrothermal conditions governed by pressure solution (*n* = 1).

Note that the creep laws mentioned above are commonly derived from compression experiments that are conducted at high temperature and pressure conditions, where the deforming material tends to be well compacted and have a relatively low porosity (<5%). However, in a frictional process, a gouge tends to be porous and deforms volumetrically to allow particles to slide, roll, and rearrange and thus accommodate shear displacement. Consequently, the local stresses transmitted across the grain contacts could be much higher than the macroscopic ones. To account for this effect, a porosity function is introduced to accompany the creep law. For the hydrothermal case where diffusion‐controlled IPS obeys a linear constitutive law (*n* = 1), we adopt a porosity function *f*(*φ*) = (*q* − 2*φ*)^−*p*^ with a *p* value of 2 (equation 4). Based on this consideration, equation 13 can be extended as $\dot{\varepsilon }\equiv B\left(T,d\right){\left[\sigma_{n}\;f\left(\varphi \right)\right]}^{n}$. To separate the effects of stress and porosity, we can rewrite the equation into

(14a) $${\dot{\varepsilon }}_{\mathrm{pl}}\equiv B\left(T,d\right){\sigma_{n}}^{n}f^{\prime \left(\varphi \right)}.$$

Here, *f*′(*φ*) is the “apparent” porosity function that accounts for the effects of both porosity and *n* value.

(14b) $$f^{\prime }\left(\varphi \right)={\left(q-2\varphi \right)}^{-M},\text{with}\;M=p\times n.$$

With the general creep law and the corresponding porosity function, we adopt the same analytical approach as in the hydrothermal case (sections 3.2.1 and 3.2.2). Table 1 summarizes the results of the frictional healing parameters derived for hydrothermal and room‐humidity conditions. For both cases, the frictional healing displays a power‐law increase with hold time. The hydrothermal results can generally be taken as a special case of the “dry” functional form with *n* = 1. Note that a power law with a small exponent can be approximated by a log‐linear law (i.e., for an arbitrary variable *x,* the relation $x^{1/n}\approx 1+\frac{1}{n}\ln\left(x\right)$ holds for a large *n* value). Therefore, in the case of large *n* values, the healing predicted for the room‐humidity conditions can be approximated by a log‐linear relation with hold time. This approximation also leads to the interpretation of frictional healing similar to that made for the slowness law: *β* is asymptotically equal to the *b* value for long hold times, as noted by previous workers (e.g., Beeler et al., 1994). We shall verify this with numerical simulations in section 4.

**Table 1:** Frictional Healing Parameters under Hydrothermal and Room‐Humidity Conditions^a^

	Hydrothermal conditions	Room‐humidity conditions (when *n* or *M* is large)	
Normalized state^b^ (*X* = *a*_*c*_ or *ψ*)	$X_{\mathrm{ss}}{\left(1+\frac{t}{\alpha }\right)}^{1/3}$	$X_{\mathrm{ss}}{\left(1+\frac{t}{\alpha }\right)}^{1/\left(M+1\right)}$	$\underset{\text{large}\;M}{\to }\frac{X_{\mathrm{ss}}}{\left(M+1\right)}\ln\left(1+\frac{t}{\alpha }\right)$
Peak healing (Δ*μ*_*pk*_)	$3b\left[{\left(1+\frac{t}{\alpha }\right)}^{1/3}-1\right]$	$b\left(M+1\right)\left[{\left(1+\frac{t}{\alpha }\right)}^{\frac{1}{\left(M+1\right)}}-1\right]$	$\underset{\text{large}\;M}{\to }b\ln\left(1+\frac{t}{\alpha }\right)$
Healing rate (*β*)	$2.3b{\left(1+\frac{t}{\alpha }\right)}^{1/3}$	$2.3b{\left(1+\frac{t}{\alpha }\right)}^{1/\left(M+1\right)}$	$\underset{\text{large}\;M}{\to }2.3b$
Cutoff heal time (*α*)^c^	*t*_*c*_	*t*_*c*_	
Limit of healing^d^	$\sim 3.5H\left(\varphi_{c}-\varphi_{0}\right)\left(1+0.74\frac{{\tilde{S}}_{0}}{\sigma_{n}}\right)$		

a In these expressions, *a*, *b*, and *D*_*c*_ are equivalent RSF parameters with physically meaningful expressions given by Chen et al. (2017) (see also Table S1). Subscript “ss” denotes prehold steady state, and *n* is power exponent of the contact creep law.

b Here, the expressions for state variable evolution apply to contact area (*a*_*c*_) and dilatancy angle (*ψ*). Porosity can be thus determined from the equation (Beeler & Tullis, 1997): tan*ψ* = *H*(*q* − 2*φ*).

c Cutoff heal time α is equal to the characteristic evolution time (*t*_*c*_ = *D*_*c*_/*V*_imp_). For short hold times, to have a more close approximation, an extra time could be added to the cutoff time such that α = *t*_*c*_+*t*_*in*_, where *t*_*in*_ is the characteristic response time from the direct effect: *t*_*in*_ = *aσ*_*n*_/(*KV*_imp_). The competing controlling deformation mechanisms distinguish between the short and long hold times and can be numerically determined from the friction‐velocity phase diagram (e.g., Figure 3c).

d The limit of frictional healing is proportional to the maximum compaction, that is, (*φ*_*c*_ − *φ*_0_). Cohesion also contributes as portioned to ${\tilde{S}}_{0}/\sigma_{n}$, where ${\tilde{S}}_{0}$ is the cohesion at grain contacts.

In the expressions given in Table 1, all the parameters are either the constitutive parameters of the operating creep mechanism (e.g., the *n* value), the fault‐related parameters (*W*, *σ*_*n*_, *K*, and *V*_imp_), or the equivalent RSF parameters (*a*, *b*, and *D*_*c*_). As given in our previous work (Chen et al., 2017), the RSF parameters can be expressed as functions of the creep‐ and fault‐related parameters (see also Table S1). All the parameters involved generally fall into two categories. One is related to the fault configuration, including shear zone thickness *W*, particle size *d*, grain packing constant *H*, critical porosity *φ*_*c*_, temperature *T*, effective normal stress *σ*_*n*_, and imposed slip velocity *V*_imp_. The other category consists of the kinetic constants of the inferred deformation mechanism(s), such as the activation energy, stress and grain size exponents (*m* and *n*), as well as strain‐rate sensitivity of gb friction, depending on the material and the in situ, temperate‐pressure‐fluid conditions. In general, all the expressions given in Table 1 can be applied to both laboratory and natural faults filled with granular material, provided that the fault structure and underlying creep mechanism(s) are well constrained, with the appropriate quantitative relations and their limitations being kept in mind. In laboratory‐simulated faults, these parameters can be directly measured from the experiments or determined from the post‐deformational samples. As applied to natural fault zones, their values can be constrained by integrating the results from laboratory experiments, field studies, and geophysical observations, as shall be briefly addressed in section 6.3.

## 4 Numerical Validation of Analytical Results

### 4.1 Model Setup of the CNS Model

The geometric model and microstructural parameters are taken the same as previously used by Chen and Spiers (2016). To compare with the analytical results (i.e., Table 1), we neglect the shear deformation in the bulk layer. Using the numerical implementation of the CNS model, we simulate SHS experiments at 80 °C and 50‐MPa effective normal stress and hydrothermal versus room‐humidity conditions.

For hydrothermal simulations, we use diffusion‐controlled IPS as the contact creep mechanism and a corresponding *p* value of 2 for the porosity function. As observed in long‐term compaction experiments, this porosity function only applies to porosities above some level *φ*_*d*ev_, below which the creep rate will deviate from the prediction by the theoretical creep laws (Niemeijer et al., 2002; Spiers et al., 2004). To account for this effect, an error function is introduced to the porosity function:

(15) $$f\left(\varphi \right)={\left(q-2\varphi \right)}^{-2}\mathrm{erf}\left(\frac{\varphi -\varphi_{0}}{\varphi_{\mathrm{dev}}-\varphi_{0}}\right).$$

Here, *φ*_0_ is a cutoff porosity (2%) corresponding to the percolation threshold for an interconnected pore network (Bryant & Blunt, 1992). This limitation only applies to fault‐normal deformation, as deformation in the shear direction does not involve volumetric strains.

For room‐humidity conditions, we adopt a functional form for the creep law as discussed in the preceding section: ${\dot{\varepsilon }}_{\mathrm{pl}}\equiv B{\sigma_{n}}^{n}f\prime \left(\varphi \right)$. Arguably, the creep rates of a “dry” gouge are much slower than for a wet gouge. We, therefore, chose a *B* value such that the compaction rate is 10^−5^ times of that in the wet case for the same *f*′(*φ*) (Niemeijer et al., 2002). As mentioned earlier, a “dry” gouge usually exhibits only a weak strain‐rate dependence, for which a stress exponent (*n* value) of 5 is taken. We use the same *p* value and cutoff porosity as for the hydrothermal case. The combination of the *p* and *n* values yields *M* = 10 for the apparent porosity function (see Figure S3). Under the limiting conditions where compaction rates are negligible, experimental results have shown that “dry” gouge has a higher steady‐state porosity than wet gouge (Chen et al., 2015; Verberne et al., 2013), possibly due to a more angular grain shape under “dry” conditions. Therefore, we use a slightly higher critical porosity for the “dry” gouge (30%). Note that the use of higher critical porosity will not change the results of frictional healing but only brings porosity to a higher reference level, which is more realistic.

### 4.2 Numerical Predictions and Comparison

We start the simulations from a situation of steady‐state sliding by setting the time derivatives of porosity and shear stress to 0. As given in Figure S4, the steady‐state frictional strength profiles predicted for both cases show consecutive transitions with increasing velocity from velocity strengthening in the plastic flow regime to velocity weakening and back to velocity strengthening in the frictional regime (as has been observed in laboratory experiments; see detailed discussion in Chen et al., 2017). In all regimes the strength of the “dry” gouge exhibits weaker strain‐rate dependencies than the wet gouge, owing to its higher *n* value. As shown earlier in Figure 3, the model predictions for the evolution of shear stress and porosity over a single SHS sequence are broadly consistent with experimental observations (see the detailed comparison in Chen & Spiers, 2016). Analogous to typical SHS experiments, we then simulate a series of SHS sequences over a wide range of hold times (10 − 10^6^ s), under both hydrothermal and room‐humidity conditions, from which the frictional healing behavior over the duration of the hold can be investigated. Representative results from the SHS simulations are given in Figure S5.

**Figure 5:**
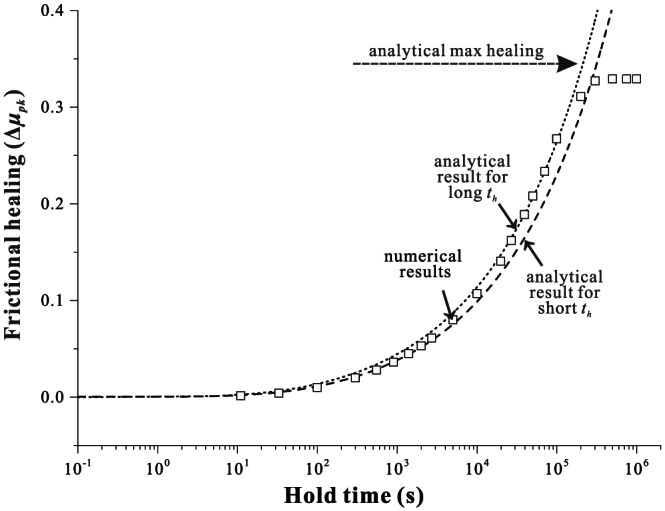
Comparison of analytical and numerical predictions of frictional healing for SHS tests under hydrothermal conditions. Two asymptotic approximations are given for short and long hold‐time end‐member cases, which correspond to hold‐time relaxation creep that is consecutively dominated by granular flow and shear creep and can be distinguished numerically from the friction‐velocity phase diagram (e.g., Figures 4 and S2). In the numerical simulations, a limit to frictional healing is predicted for long hold times.

**Figure 6:**
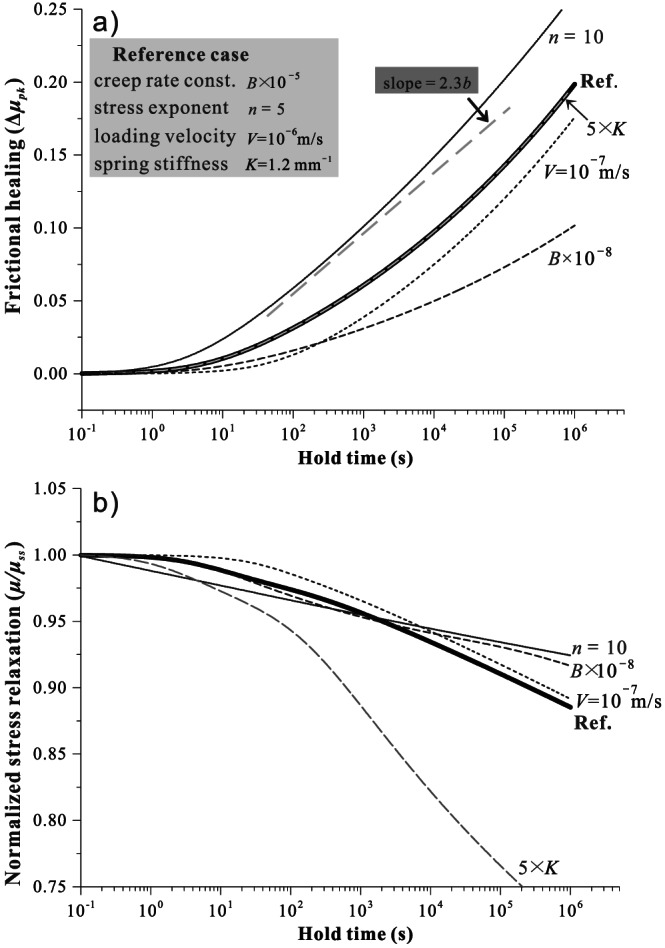
Numerical results of (a) frictional healing and (b) normalized stress relaxation for SHS tests simulated at room‐humidity conditions. The same model settings are adopted as for the hydrothermal case, except that here, the contact creep follows a power law with a stress exponent of 5 and with a kinetic constant that is five orders of magnitude lower, defining the “dry” reference case. Parametric analyses of other factors, as indicated in the legend and illustrated by the dashed lines, are performed with respect to the reference case. For large *n* values (~10), the healing rate asymptotically approaches the *b* value by a factor of ln (Brzesowsky et al., 2014).

Under hydrothermal conditions, the prediction of peak frictional healing from SHS simulations follows a power‐law increase with hold time (squares in Figure 5), showing good agreement with the results from the two analytical expressions, as derived for both short and long hold times (see the two dashed lines that are separated by a few multiples of *t*_*c*_). The numerical results also predict a limit to the attainable magnitude of healing at hold times greater than 2·10^5^ s. This saturation in frictional healing occurs when the gouge compacts to the cutoff porosity, resulting in a maximum peak friction coefficient of about 0.95. Under room‐humidity conditions, the frictional healing shows the characteristic “Dieterich‐type,” log‐linear relations with hold time (see the reference case in Figure 6a). Again, the result is consistent with the prediction from analytical expression. Unlike the hydrothermal case, the saturation of healing is not reached for the hold times investigated (<10^6^ s).

To further investigate the sensitivity of the prediction, we vary a number of key parameters that are currently not well constrained at room‐humidity conditions, that is, the creep kinetics (*B*), the stress exponent (*n*), and variables that can be experimentally controlled, that is the reshear velocity (*V*_imp_) and stiffness (*K*). As shown in Figure 6a, with smaller *B* value, the gouge tends to have slower frictional healing and a similar or slightly shorter “apparent” cutoff time. With larger *n* values, the curve of healing versus log (*t*_*h*_) becomes more linear, accompanied with a shorter “apparent” cutoff time. As the loading velocity decreases, the cutoff time increases and the amplitude of healing decreases, with negligible changes in the slopes (Figure 6a). In the present framework, a higher stiffness does not affect the magnitude of healing but apparently will cause faster relaxation (Figure 6b). These trends predicted are all consistent with results obtained from previous experiments performed at room‐humidity conditions (Beeler et al., 1994; Marone, 1998; Marone & Saffer, 2015; Richardson & Marone, 1999).

## 5 Model Validation: Frictional Healing Explained by Independent Compaction Data

According to the analytical expressions derived (Table 1), the frictional restrengthening achieved over a hold time *t*_*h*_ can be calculated as

(16) $$∆\mu_{\mathrm{pk}}\left(t_{h}\right)=b\left(M+1\right)\left[{\left(1+\frac{t_{h}}{\alpha }\right)}^{\frac{1}{\left(M+1\right)}}-1\right].$$

This equation indicates that the frictional healing behavior (i.e., log‐linear versus power‐law growth) is essentially controlled by the rheological parameters that govern the compaction creep of the gouge, the *M* value in particular (equations 14a and 14b). To be rigorous in the microphysical sense, one could estimate all the parameter values in equation 16 based on theoretical considerations and laboratory observations. While the CNS model invites for such an approach by considering the exact deformation mechanism, grain size, and geometric factors (among other parameters), it is in practice challenging to perform such a model validation through quantitative comparison of equation 16 with laboratory measurements of frictional healing. This is partly due to the large epistemic uncertainties associated with the (possibly unknown) creep mechanism(s). To largely circumvent the uncertainty in the creep behavior, we choose to fit the equations 14a and 14b to independent laboratory compaction data to get the *M* value. The chosen compaction experiments were performed on the same or similar materials as the SHS friction experiments under similar conditions. While this fitting method does not validate the full model, it allows us to verify the important “frictional” component of the model, that is, the time‐dependence of frictional healing associated with gouge densification. Recently, using an empirical creep law derived from compaction data under 100 °C and brine‐saturated conditions, Hunfeld et al. (2019) have reproduced their velocity‐stepping tests for a carbonate‐anhydrate‐rich gouge sheared at the same conditions. Future work should include better characterization of the relevant creep mechanism. From theory, the *b* value as appearing in equation 16 can also be calculated from the creep law and from a few other known constants (Chen et al., 2017). However, due to the uncertainty and also because of the different *d*, *T*, and *σ*_*n*_ that were used in the two sets of experiments (friction vs. compaction), the *b* and *α* values are instead fitted to the frictional healing data to get an estimate for the magnitude of frictional healing. We later assess the validity of the obtained parameter values by comparing with values reported in literature. The main objective is to predict the correct time evolution of frictional healing (i.e., the shape of the healing curve), which is primarily controlled by the *M* value. Since the *M* value is constrained by independent compaction experiments, this approach facilitates testing of our hypothesis that the difference between log‐linear and power‐law healing can be explained by the dominant creep mechanism (and thus variations in the corresponding *M* value). With the shape of the healing curve being fixed by the compaction experiments, the *b* and *α* values only dictate the amplitude of healing.

**Figure 7:**
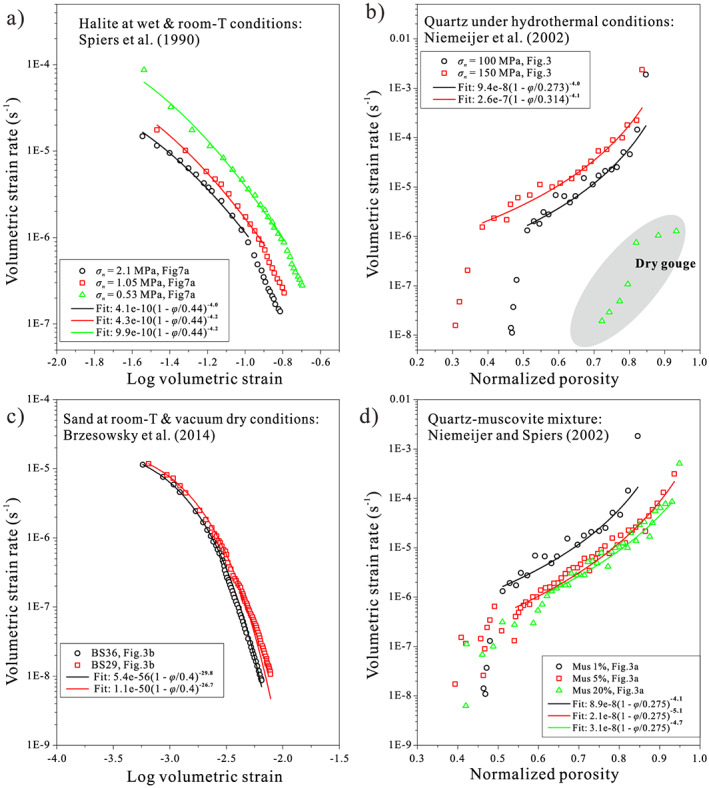
Laboratory compaction data and the fitted creep laws for (a) wet halite, (b) quartz at hydrothermal condition, (c) quartz at room‐humidity condition, and (d) quartz‐muscovite mixtures. The results are plotted against either “log volumetric strain” or “normalized porosity,” following the original plots from the literature. In case (b), data from the “dry” gouge are added for comparison. The fitted “creep law” is rewritten from the equation (Chen et al., 2015): ${\dot{\varepsilon }}_{\mathrm{pl}}\equiv C{\left(1-\varphi /\varphi_{c}\right)}^{-M}$, where *C* = *B*(*T*,*d*)*σ*_*n*_^*n*^(2*φ*_*c*_)^−*M*^.

**Figure 8:**
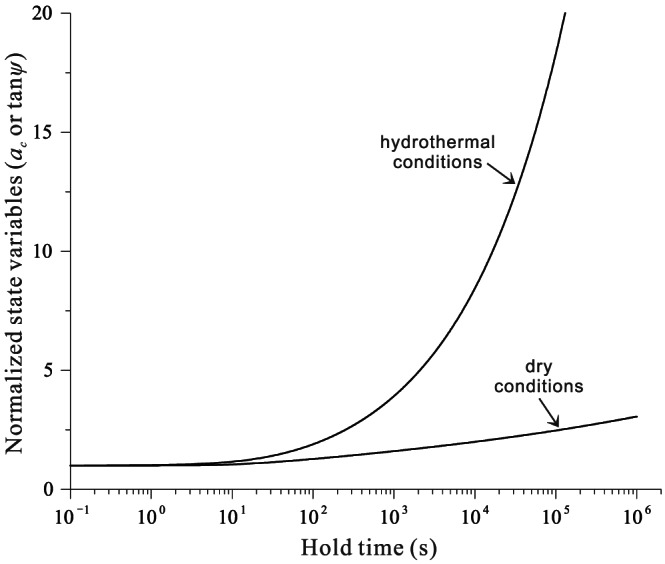
Evolution of normalized microstructural state (average contact area or dilatancy angle) with respect to the logarithm of hold time, simulated for SHS tests under hydrothermal versus room‐humidity conditions. The results are from the same tests simulated in Figure 5 and the reference case in Figure 6.

We apply the above approach to halite under brine‐saturated conditions (Niemeijer et al., 2008), quartz under hydrothermal conditions (Nakatani & Scholz, 2004), and (quartz) sands under room‐humidity conditions (Marone, 1998) and finally to natural fault rocks (Ikari et al., 2016). As shown in Figure 7, laboratory compaction data are usually presented as volumetric strain rate ( $\dot{\varepsilon }$) plotted against porosity *φ* or logarithmic volumetric strain log(*ε*), with the relation *ε* = *φ*/(1 − *φ*). We first fit these data with our modified creep law for porous material (equations 14a and 14b), which, for the fitting convenience, can be rewritten as ${\dot{\varepsilon }}_{\mathrm{pl}}\equiv B\left(T,d\right){\sigma_{n}}^{n}{\left(1-\varphi /\varphi_{c}\right)}^{-M}$, where *φ*_*c*_ is porosity at the “touch” point of the experiment (i.e., the porosity before any stress is applied). This fitting gives us an “apparent” creep law with two independent variables, *B*(*d*,*T*)*σ*_*n*_^*n*^, and *M* value. The compaction data and the fitted creep laws are given in Figures 7a–7d, respectively, where the dry data indicate a higher *M* value (~29) than the wet data (4–5.7). Note that these apparent *M* values for the wet samples are higher than the theoretical values for IPS‐dominated compaction creep (1–4), which possibly results from the deceleration of compaction rates as seen in long‐term compaction tests (e.g., Spiers et al., 2004). For all these cases tested, the predictions for both wet and dry frictional healing behavior agree favorably with the laboratory friction data (Figures 8a–8d). Specifically, the frictional healing behavior displays different relations with hold time, that is, “log‐linear” versus “stronger than log‐linear,” consistent with both the analytical and numerical results. The *b* values obtained for all the cases tested fall in the range from 0.001 to 0.034, which are comparable with previous experimental results (e.g., Carpenter et al., 2014; Renard et al., 2012). A similar agreement is also seen in the α values, which fall between 0.1 and 350 s, and mostly in the 1‐ to 10‐s range.

Surprisingly, the frictional healing of natural fault rocks at room temperature conditions (Ikari et al., 2016) can be moderately well explained by the compaction data of quartz‐muscovite mixtures under hydrothermal conditions (Figures 7d and 8d). This agreement is not immediately expected, as the dominant compaction mechanism inferred for the quartz‐muscovite mixtures (pressure solution creep) likely differs from the deformation mechanism(s) operating in the phyllosilicate‐rich gouges at room conditions. Moreover, the present model is not directly applicable to phyllosilicate‐rich materials, and alternative models such as the one proposed by Bos and Spiers (2000) are more appropriate. We resolve this apparent paradox by noting that by comparing the *M* value obtained from the compaction data with the shape of the frictional healing curve, we only constrain the porosity dependence of the dominant deformation mechanism(s). Second, the consistency between the compaction and healing experiments can be interpreted more broadly at a conceptual level; even though the CNS model does not capture the details of the microstructure of phyllosilicate‐rich gouges, the fundamental notion that frictional healing originates from compaction may still hold (as observed in previous studies; e.g., Nakatani, 1998; Richardson & Marone, 1999; Niemeijer et al., 2008). In conclusion, the good agreement between the frictional healing data and the model predictions based on independent compaction experiments testify to a broad validation of the underlying principles of the proposed model.

## 6 Discussion

### 6.1 DH and NDH Unified by the Microphysical Model

We have shown that the CNS model predicts distinct frictional healing behavior when distinguishing contact creep laws are used. The results reproduce the different healing behaviors that have been observed in friction experiments under hydrothermal and room‐humidity conditions (Figures 4 and 5).

With diffusion‐controlled IPS as the creep mechanism, the frictional healing displays a power‐law increase with time, rather than with log (*t*_*h*_), with an exponent of 1/3 (Table 1). This exponent is expected to vary between 1/5 and 1/2, depending on the rate‐controlling process, that is, dissolution, diffusion, and precipitation (*M* value in the range of 1–4; Spiers et al., 2004). Healing stronger than log‐linear relation has been observed in previous experiments on quartz (Nakatani & Scholz, 2004; Yasuhara et al., 2005), calcite (Chen et al., 2015), and halite (e.g., Niemeijer et al., 2008; Renard et al., 2012). In these studies, the enhanced healing was inferred to be result of the growth in contact area by either IPS or subcritical crack growth, with exponents of 0.24–0.67, consistent with our model predictions. The power‐law relation is also predicted for the growth of contact area: $a_{c}=a_{c0}{\left(1+\frac{t_{h}}{\alpha }\right)}^{1/3}$(Table 1), which has been observed in situ on halite‐halite and quartz‐quartz contacts undergoing fluid‐assisted healing process (Beeler & Hickman, 2015; Hickman & Evans, 1992). Moreover, the cutoff time (*α*) observed in the frictional experiments falls in the range 50–5,000 s (e.g., Bos & Spiers, 2002; Nakatani & Scholz, 2004; Niemeijer et al., 2008). A similar “apparent” cutoff time of ~1,000 s can also be obtained from our model prediction as plotted in semilogarithmic space, although the result is essentially conforming to a power law with an intrinsic cutoff time of 17 s.

**Figure 9:**
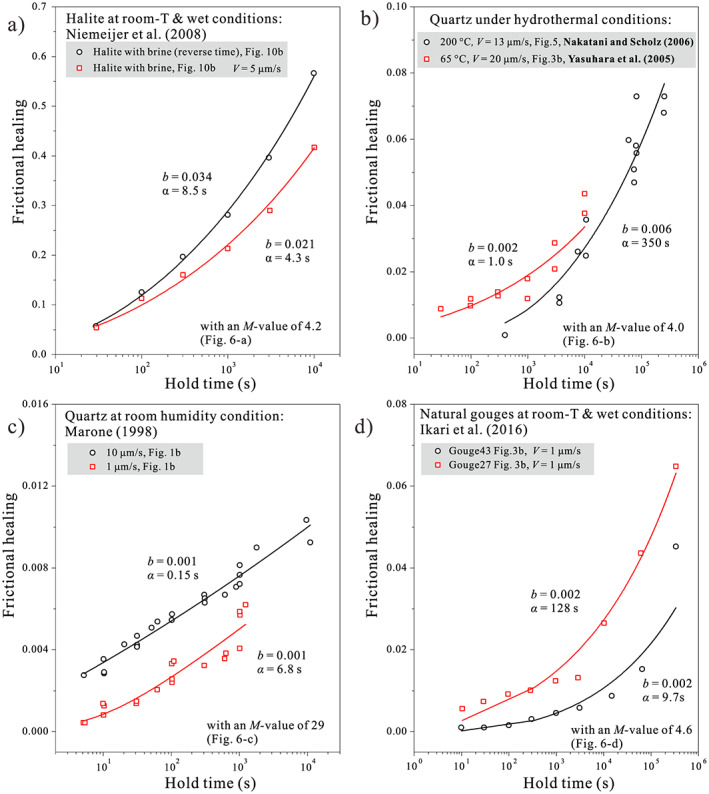
Prediction of frictional healing with hold times for (a) wet halite, (b) hydrothermal quartz, (c) room‐humidity quartz, and (d) natural fault gouges, in which the kinetic parameters were obtained from independent compaction experiments that are performed at similar conditions. The squares and circles in (a)–(d) are laboratory frictional healing data. The corresponding compaction data are given in Figures 7a–7d, respectively. The frictional healing results (in solid lines) are calculated from equation 16. In each case, the parameters used are labeled, where the *M* value is derived from the compaction data, and the *α* and *b* values are best‐fitted results.

As seen in the numerical and analytical results (Figure 6; Table 1), frictional healing under room‐humidity conditions also exhibits a power‐law growth with time, but it can be closely approximated by a log‐linear relation. The healing rates predicted display the same magnitude as the *b* value (for large *n* value), which has been well documented by previous experimental studies performed at dry and low‐temperature conditions (e.g., Marone, 1998; Marone & Saffer, 2015). For the present model settings, the predicted “apparent” healing rates fall in a reasonable range (0.013–0.13 decade^−1^; Figure 6). The approximate log‐linear relation also applies to the growth in contact area, that is, $a_{c}\approx a_{c0}\left[1+\frac{1}{\left(M+1\right)}\ln\left(1+\frac{t_{h}}{\alpha }\right)\right]$ (Table 1; Figure 9), which is consistent with the in situ optical observations, although these results are limited to bare surfaces (e.g., Dieterich & Kilgore, 1996). In contrast to the hydrothermal case, the “apparent” cutoff time for the “dry” gouge is closer to the intrinsic *α* value (4–10 s, Figure 5). Note that the same parameters are employed for both hydrothermal and room‐humidity conditions, except that we use slower kinetics (smaller *B* value) and a larger *M* value for the rheology of the “dry” gouge. At present, the magnitude of *M* (=*n* × *p*) is unconstrained by a particular known creep mechanism, but large *M* values are warranted by the weak strain‐rate dependence of contact “creep” (large *n* value) facilitated, for example, by subcritical cracking.

Recently, Ikari et al. (2016) performed SHS friction experiments under room temperature and wet conditions on samples retrieved in fault drilling campaigns (Figure 8d). They found that for short hold times, frictional healing follows a log‐linear dependence on hold time, indicating small healing rates. However, over a much larger span of hold times (up to ~350,000 s), the healing rates accelerate, and the frictional healing is better described by a power‐law relation. These experiments, in the light of the CNS model, imply that the DH and NDH behaviors share the same physical origin, though the dominance of one type of healing behavior depends on the time span investigated. We further propose that NDH is promoted under hydrothermal conditions since under these conditions, IPS is the most efficient creep mechanism (Frost & Ashby, 1982), which can generate significant state changes within the time span of a typical experiment. Conversely, under room‐humidity conditions, the contact rheology tends to have slower kinetics and larger *n* value, and as such, the state change becomes less sensitive to hold time, making a power‐law healing appear as log linear (DH) over the range of hold times employed in laboratory tests (10^1^–10^4^ s). Moreover, the geometrical setup of the present model is in principle applicable to roughly equigranular materials (Figure 2). Alternative models such as the one proposed by Bos and Spiers (2000; hereafter referred to as the B&S model) are required to capture key features of phyllosilicate‐rich gouges, such as the existence of a through‐going foliation. Nonetheless, as these models share a similar physical origin, the general conclusions drawn from the CNS model likely hold in the framework of the B&S models, offering some confidence that our interpretations of granular gouges apply at least qualitatively to phyllosilicate‐bearing samples (e.g., to natural fault gouges; Figure 8d). Finally, we note that numerous previous studies in which bare fault surfaces were used demonstrated log‐linear fault healing. Our model cannot directly be applied to such faults because the model was developed for friction of a granular gouge exhibiting a mature structure (i.e., constant nominal grain size, grain shape, and shear band thickness), whereas in bare surface experiments, the amount of gouge being produced, its particle size distribution, and its degree of localization are still evolving. Further investigation is required to extend the model applicability to less mature faults, incorporating the evolution of wear rate, grain size, and shear zone thickness.

### 6.2 Microphysical Interpretation of Frictional Healing and its Limit

In addition to offering a unified explanation for DH and NDH behaviors, the CNS model relates the frictional healing to volumetric strains of the granular sample for which a clear correlation has been observed in numerous laboratory experiments (Bos & Spiers, 2002; Richardson & Marone, 1999). For instance, the logarithmic time dependence of compaction has been previously observed in SHS experiments (Beeler & Tullis, 1997; Carpenter et al., 2016), and empirical models have been constructed to incorporate gouge compaction with existing RSF laws (Segall & Rice, 1995; Sleep et al., 2000). In the CNS model, porosity is taken as the microstructural state variable, and frictional healing is attributed to the compaction achieved during a hold. The corresponding maximum healing can be calculated as

(17a) $$\max\left(∆\mu_{\mathrm{pk}}\right)=\max\left(\Delta \varphi \right)\frac{\partial \mu }{\partial \varphi }=2H\left(\varphi_{c}-\varphi_{0}\right)\frac{1+{\tilde{\mu }}^{2}}{{\left(1-\tilde{\mu }\tan\psi_{\max}\right)}^{2}}.$$

Assuming a carbonate fault gouge ( $\tilde{\mu }$ = 0.6 and tan*ψ*_max_ = 0.2), the maximum healing is approximated as

(17b) $$\max\left(∆\mu_{\mathrm{pk}}\right)\approx 3.5H\left(\varphi_{c}-\varphi_{0}\right).$$

This relation is verified by the numerical results for the hydrothermal case (Figure 5). Our model predicts a maximum frictional strength of 0.95, which is interestingly consistent with the peak strength measured by Carpenter et al. (2014) for refracturing (and shearing) of an intact carbonate fault surface under water‐saturated conditions (their “SS/Cc” configuration indicating that a slip surface is sheared against cataclasite), although their experiment was performed at low normal stress, where other factors such as cohesion, as discussed later, might have a significant contribution.

As the grain‐to‐grain contact area is related to the porosity through the porosity function in the CNS model, a correlation can be made between fault strength and contact area (Table 1). To some extent, this is consistent with the classic adhesive theory of friction that frictional healing is proportional to the growth in real contact area (*μ* ∝ *a*_*c*_; e.g., Bowden & Tabor, 1964; Estrin & Bréchet, 1996). However, the classic friction models (e.g., RSF laws) assume the macroscopic frictional strength to be a direct consequence of the size of contact area (i.e., Δ*μ* ∝ Δ*a*_*c*_), while in the CNS model, the friction strength of a granular gouge consists of two components ( $\mu \approx \tilde{\mu }+\hat{\mu }$, as approximated from equation 1c, where $\hat{\mu }$ is dilatant strength that $\hat{\mu }\equiv \tan\psi$, and $\tilde{\mu }$ is the gb sliding strength; Chen et al., 2017), and it is only the strength increase due to a decrease in porosity (compaction) that is responsible for healing ( $\Delta \hat{\mu }\propto ∆\varphi$). From the model geometry, this decrease in porosity correspondingly results in the growth of contact area (see their relation in equation 2b). Hence, frictional healing and contact area growth in fault gouges are also correlated in time ( $\Delta \hat{\mu }\propto \Delta a_{c}$). In other words, in the CNS model, contact area change only occurs concomitant with porosity change, in contrast to the RSF laws in which contact area can evolve without directly affecting porosity. Moreover, in previous models for bare rock interfaces, the growth in contact area is considered to result from pure creep of contact asperities, with the exception of recent studies that consider the effects of asperity elasticity (e.g., Perfettini & Molinari, 2017). In contrast, the CNS model ties the increase in contact area to volumetric reduction of the deforming gouge (Richardson & Marone, 1999).

While sharing a similar physical origin, the microscopic mechanisms that control healing are, nevertheless, different between different testing conditions. Under hydrothermal conditions where IPS dominates, continued compaction occurs over the hold period, during which, grain rearrangement will be readily involved, causing the increase in apparent overlapping area between two grains (*A*_*c*_, as illustrated in Figure 2c). By contrast, for a “dry” gouge, the “creep” rate is slow. As such, fast, time‐dependent compaction is merely attributed to granular slip, which diminishes in a very short time. Presumably, the increase in contact area will be mostly due to the increases in *A*_*l*_, that is, the growth of individual asperity (Figure 2d). This process is similar to the interpretation of frictional healing based on RSF laws, which actually explains why a log‐linear healing can be derived from the CNS model for the “dry” case (Figure 6).

So far, we have assumed that frictional healing is entirely governed by time‐dependent compaction. Under hydrothermal conditions, mineral dissolution, precipitation, and the so‐called cementation process may generate cohesion (e.g., Muhuri et al., 2003; Tenthorey & Cox, 2006; van den Ende & Niemeijer, 2019). As a first‐order approximation, we assume grain‐scale cohesion $\tilde{S}$ to be proportional to the growth in contact area. With the contribution from cohesion, the limit of friction healing becomes (see derivations in Appendix A):

(18a) $$\max\left(∆\mu_{\mathrm{pk}}\right)\approx 2H\left(\varphi_{c}-\varphi_{0}\right)\frac{1+{\tilde{\mu }}^{2}+\frac{{\tilde{S}}_{0}}{\sigma_{n}}}{{\left(1-\tilde{\mu }\tan\psi_{\max}\right)}^{2}}.$$

Compared with equation 17a, equation 18a contains an extra term ${\tilde{S}}_{0}/\sigma_{n}$, where ${\tilde{S}}_{0}\;$ is the normalized cohesion strength at grain contacts (in unit of Pa). Again, using typical values for a carbonate gouge, equation 18a becomes

(18b) $$\max\left(∆\mu_{\mathrm{pk}}\right)\approx 3.5H\left(\varphi_{c}-\varphi_{0}\right)\left(1+0.74\frac{{\tilde{S}}_{0}}{\sigma_{n}}\right).$$

This relation gives the maximum frictional healing caused by both compaction and cohesion at grain contacts. Multiplying it by effective normal stress (*σ*_*n*_) gives the absolute fault restrengthening (in units of Pa). As it becomes apparent (and as is reasonably expected), the contribution of cohesion to the increase in the apparent friction strongly depends on effective normal stress. With increasing depth for a natural fault, its effect becomes less significant, assuming that the effective normal stress continuously increases with depth. At shallow depths or at greater depths but with overpressurized fluids, cementation may exert a significant contribution to the restrengthening. Due to the low effective normal stress that may limit the rate of compaction (and thus growth in contact area), the resulting frictional healing may not show a power‐law healing as expected for hydrothermal conditions.

### 6.3 Implications for Natural Fault Restrengthening

**Figure 10:**
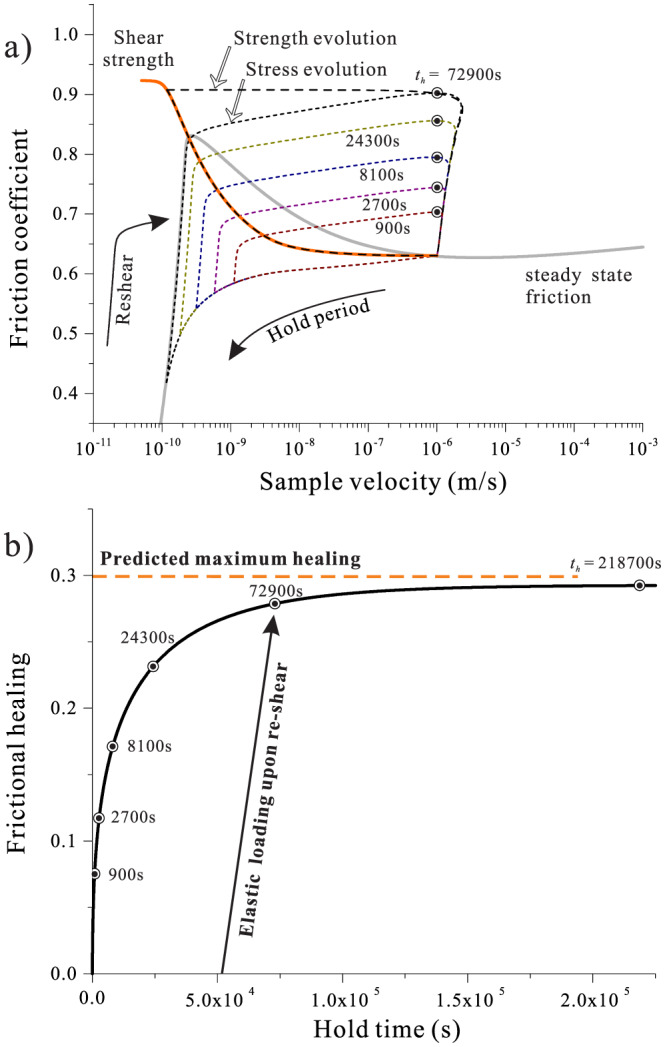
(a) Friction‐velocity phase diagram illustrating laboratory SHS sequences with different hold times. The steady‐state friction/flow strength profile is added in a solid gray line. As illustrated by the event with a hold time of 72,900 s, failure occurs at the point where the shear stress intersects with the shear strength of the fault (in the dashed line). (b) Evolution of frictional strength against the duration of the interseismic period as extracted from (a), with the limit of frictional healing reached after about 3·10^5^ s.

SHS experiments are assumed to be laboratory analogs to earthquakes cycles. Here, our physically based model offers new perspectives for the seismic cycle behavior of a natural fault, which will be briefly discussed in this section. Figure 10 presents the simulation results for the evolution of frictional strength and stress during SHS tests with various hold times. As stress relaxes after a hold, the fault will creep with decreasing rates and finally evolve along the steady‐state plastic flow profile (Figure 10a). Upon reshear, shear stress increases, and failure occurs when shear stress meets the shear strength of the gouge (as illustrated by the event with hold time of 72,900 s). However, the predicted power‐law growth in the peak friction and the shear strength of the fault cannot hold indefinitely (Figure 10b), as eventually porosity reaches a terminal value. Neglecting other contributions such as cohesion, the limit in frictional restrengthening is therefore controlled by the terminal state of compaction (equations 17a and 17b; Figure 10b).

Inspired by the numerical analog results and based on the relations derived in this work, we extrapolate the laboratory results of Chen et al. (2015) to the temporal scales typical for a natural carbonate fault subjected to similar conditions, under which the same microphysical, rock deformation processes operate as assumed throughout this work, that is, granular flow and pressure solution creep (Smeraglia et al., 2017; Tesei et al., 2013). To avoid the structural and compositional complexities of a natural fault and for illustration purposes, in this extrapolation, we focus on the timing of frictional restrengthening of a hypothetical fault patch (or asperity) in a carbonate fault at an average depth of ~4 km, corresponding to the experimental conditions (*T* = 80 °C and *σ*_*n*_ = 50 MPa). The question of strain accommodation during various stages of a seismic cycle is intriguing and unfortunately not well constrained. In both natural and experimental carbonate‐bearing faults, it is commonly seen that extremely localized principal slip zones or “surfaces” (PSS) consisting of nanocrystalline particles are developed within or at the boundaries of the ultracataclasite zones that are composed of particles of micron meter in grain size (e.g., De Paola et al., 2015; Fondriest et al., 2013). However, it is not yet clear if it is the ultracataclasite zone or the PSS that is representative for the deformation during the nucleation or interseismic phase of an earthquake. Since extreme localization may have occurred and generated the PSS during coseismic slip (Smith et al., 2015), the ultracataclasite zones are more likely to be involved during the nucleation phase (e.g., Tesei et al., 2013). In accordance with field studies describing the internal structure of natural carbonate faults such as those in Central and Northern Apennines, Italy (e.g., the Tre Monti Fault Zone, Smith et al., 2011; the Spoleto Thrust Fault, Tesei et al., 2013), we assume that interseismic fault deformation primarily occurs in an ultracataclasite shear zone of 5 mm in thickness, with a mean grain size of 20 μm (refer to figure 3 of Smith et al., 2011 or figure 5 of Tesei et al., 2013). To explore the sensitivity of the results to these choices, we will vary the shear zone thickness and mean grain size and also investigate the scenario that the interseismic deformation localizes into the PSS, for which we assumed a 50‐μm thickness and 100‐nm mean grain size. As mentioned earlier, fault zones may be filled with overpressurized fluids. To investigate this, we also simulate a case at low effective normal stress (*σ*_*n*_ = 10 MPa). Since the fault spends little time sliding at high slip rates during the post seismic period, we use a (sub)seismic slip rate of 100 μm/s as the prehold slip rate, which produces an initial porosity close to the critical value (*φ*~*φ*_*c*_). Natural faults are highly heterogeneous in their composition, shear zone width, and in situ effective normal stress conditions, so adopting the aforementioned simplifying assumptions only provides first‐order, qualitative insights into the seismic cycle behavior.

**Figure 11:**
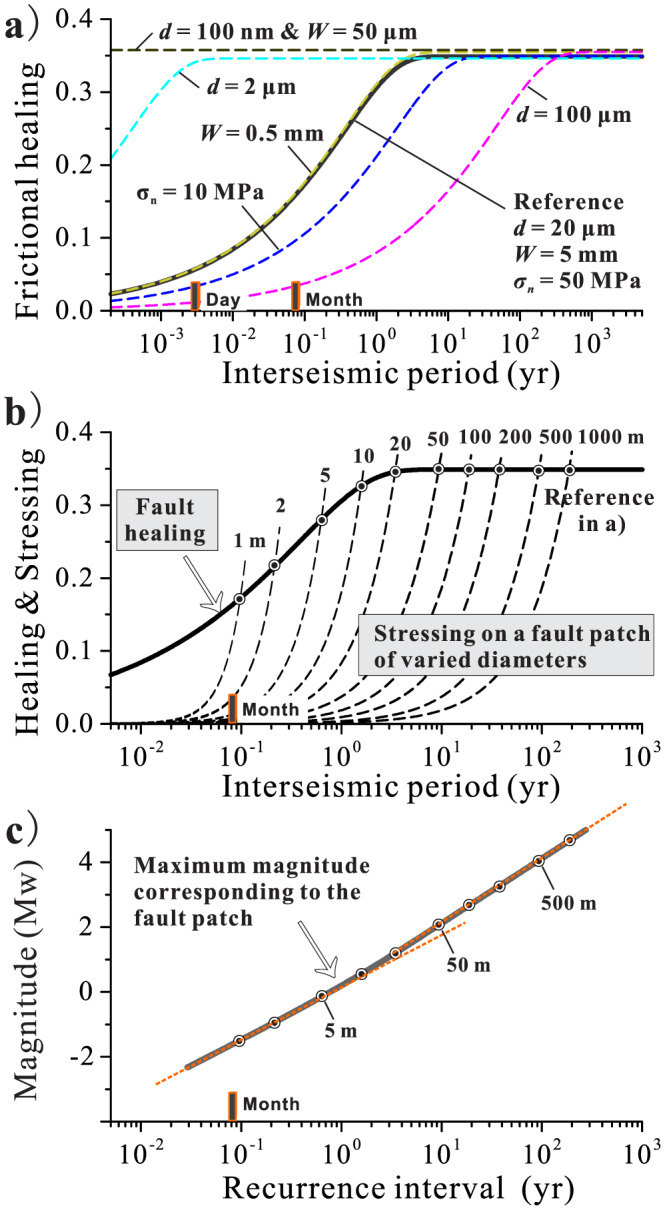
Extrapolation of the laboratory results to a hypothetical fault patch (asperity) in a natural carbonate fault at an average depth of 4 km (*σ*_*n*_ = 50 MPa and *T* = 80 °C). The parameters used are the same as for the laboratory simulated fault, except for a more realistic internal fault structure for natural faults (i.e., shear zone thickness and mean grain size; see detailed explanation for the parameters chosen in the text). (a) Predicted frictional healing as a function of interseismic period. (b) Predicted earthquake recurrence time and (c) maximum magnitude for a carbonate fault patch with varied diameters (1–1,000 m). Parametric analyses were performed in (a) with respect to the reference parameters to investigate the effects of mean grain size *d*, shear zone thickness *W*, and effective normal stress *σ*_*n*_. A natural earthquake is expected to occur when shear stress meets the strength. Maximum magnitudes of the potential earthquakes from the simulated fault patches are estimated using the seismic magnitude‐moment relation (Kanamori, 1977).

With these parameter values as the reference (*T* = 80 °C, *σ*_*n*_ = 50 MPa, *d* = 20 μm, and *W* = 5 mm), the recovery of shear strength of the fault after an earthquake follows a power‐law relation and approaches the healing limit of ~ 0.35 after 4 years (see the solid black lines in Figure 11a). An increase in grain size or a decrease in effective normal stress will cause slower frictional healing. Varying the shear zone thickness does not change the healing rate but will slightly increase the limit of frictional healing due to a lower steady‐state friction during the preceding sliding period (i.e., Δ*μ* = *μ*_pk_ − *μ*_ss_). In the case that deformation occurs in the nanocrystalline PSS (*d* = 100 nm and *W* = 50 μm), our result shows that the fault can heal completely within 2 hr after an earthquake (Figure 11a). This result suggests that after an earthquake, the nanocrystalline PSS could strengthen much faster than the remainder of the fault (e.g., the surrounding ultracataclasite, Figure 11a). There is no evidence that the nanoparticles in PSS are weak, at least at nucleation slip rates (e.g., Verberne et al., 2014), so that it is unlikely slip remains localized there throughout much (all) of the seismic cycles.

To predict the earthquake recurrence time, the stressing of the fault during the interseismic period is estimated as *KV*_plate_ *t*/*σ*_*n*_. Here *V*_plate_ is the far‐field (tectonic) loading rate taken to be 5.0 mm/year (which we set to be slightly larger than the measured interseismic velocity of 2 mm/year; Benedetti et al., 2013), and *K* is the effective stiffness of a circular crack calculated as $K=\frac{E}{2\left(1-v^{2}\right)L}$, where *L* is the diameter of the asperity (or fault patch) and *E* and *v* are Young's modulus and Poisson's ratio of the surrounding rocks (35 GPa and 0.25), respectively. Since we assume a fixed hypocenter depth at ~4 km, we only simulate small fault patches of less than 1,000 m in diameter, as to minimize the influence of the depth dependence of the in situ conditions. As indicated by the intersections between shear strength and stress evolution trajectories (the circles in Figure 11b), the model predicts recurrence times of 0.5, 9, and 90 years for fault patches of 5, 50, and 500 m in diameter, respectively.

The seismic moment *M*_0_ released from a fault patch can be calculated as *M*_0_ = *∆τ* × *A*^3/2^, where *A* is the area of the patch and *∆τ* is the stress drop. Since the average stress drop during a rupture process is usually smaller than the strength drop due to stress concentration at the crack tip, the restrengthening predicted by our model (*∆τ*_pk_ = *∆μ*_pk_ × σ_*n*_) can only be used as an upper bound of *∆τ*. Using the seismic moment‐magnitude relation $M_{w}=\frac{2}{3}\log_{10}M_{0}-6.06$ (Kanamori, 1977), we can further estimate the earthquake moment magnitudes. Since this approach only predicts the maximum earthquake magnitudes, Max (M_w_), that could potentially recur on these patches, we thus focus on the relative magnitude change rather than the absolute level. As given in Figure 11c, the results predict Max (M_w_) of ~0.0, 2.0, and 4.0 for these three patches (5, 50, and 500 m in diameter), respectively. Within the sizes of fault patches investigated (1–1,000 m), the predicted Max (M_w_) shows a linear relation with the logarithmic interseismic time (or recurrence interval), except for a small kink when reaching the healing limit (indicated by the two dashed lines; Figure 11c). A linear relation between relative seismic moment and recurrence interval was also obtained from a natural fault system by analyzing the repeating earthquakes after an earthquake (Marone et al., 1995; their CA1 sequence consisting of 19 events that followed the 1984 M6.2 Morgan Hill earthquake). Note that these repeaters are small magnitude earthquakes (M1.4–1.6) that are believed to rupture the same fault patch at a depth of ~6 km (Marone et al., 1995). Linking seismic moment to stress drops, these authors further estimated the fault healing rate (depicted by the average slope in the stress drop‐recurrence interval diagram) using a logarithmic relation derived from the classical rate‐and‐state friction laws. Motivated by our extrapolation results (Figures 11b and 11c), we revisited the results reported by Marone et al. (1995; Table S2) and found that the data of stress drop against recurrence interval can be described by a power‐law relation equally well (Figure S6). Fitting the data gives a power‐law exponent of 0.328 (±0.07), which is incidentally consistent with our theoretical value of 1/3, with pressure solution to be the dominant creep (healing) mechanism. Detailed studies based on the CNS model that incorporates the realistic fault structure, the in situ temperature‐pressure‐fluid conditions, as well as the fault rock composition at the focus depths of the repeating earthquakes are warranted in the future.

Even though the present extrapolation is relatively crude, it demonstrates a simple approach for a microphysically based prediction of earthquake recurrence, which enables the incorporation of laboratory and field observations. A microphysically based model can offer an alternative for the interpretation of laboratory mechanical and (micro)structural data and represents the mechanics of natural faults more accurately. Note that the present model is only applicable for small earthquakes involving single, small fault patches (<1 km), beyond which the scaling relations between earthquake magnitude, fault patch size, and recurrence interval are expected to be different from those obtained in the present model (Figures 11b and 11c), especially for large earthquakes that could rupture multiple fault asperities (patches), generating long rupture lengths (Mai & Beroza, 2000). Future work on predicting fault restrengthening of a specific fault zone should include the implementation of the microphysical model into numerical earthquake cycle simulators (e.g., van den Ende et al., 2018), which will allow for capturing the complexity exhibited by natural faults.

## 7 Conclusions

In the present study, we investigate the restrengthening of a gouge‐filled fault zone in the framework of the CNS model, which essentially links the macroscopic friction behavior of a granular material to the shear zone deformation processes operating in the microscale. Our results help resolves the long‐existing debate of logarithmic versus power‐law frictional healing as observed in laboratory experiments and also illuminate the relevant conditions. We find that by considering the differences in grain contact creep rheology that may operate under certain conditions, both types of healing can be predicted by the model. Under hydrothermal conditions, healing exhibits a power‐law dependence on hold time, with an “apparent” cutoff time of hundreds of seconds. Under room‐humidity conditions, where contact creep deformation exhibits only a weak strain‐rate dependence, the predicted healing still possesses a power‐law evolution with hold time, but it can be well approximated by a log‐linear relation over laboratory time scales with a cutoff time of a few seconds. Numerical implementation confirms the predictions, with a broad agreement with previous laboratory observations.

Under boundary conditions corresponding to typical SHS tests, we derive analytical expressions for frictional healing parameters, for example, frictional healing rate and intrinsic cutoff time. Without cohesion, the limit in frictional restrengthening is controlled by the terminal state of compaction of the gouge. The predictions from these expressions are fully consistent with the numerical implementation. Based on the analytical results, we further demonstrate that the diverse healing behavior observed for different materials can in general be explained by independent compaction data of the material, testifying to high validity of the model. Gaining insights from these consistencies, we extrapolate the model to a fault patch (asperity) on a natural carbonate fault zone at a given depth and stressing conditions. Future work should include the implementation of the microphysical model and fault heterogeneities into numerical earthquake cycle simulators to capture the complexity exhibited by natural faults.

## Supporting information



Supporting Information S1

## Appendix A Limit of Frictional Healing (With Presence of Cohesion)

As measured from a series of friction tests at different normal stresses, the macroscopic cohesion is typically in the range of 0.1–1 MPa (Ikari et al., 2013; Ikari & Kopf, 2011), but its value increases enormously at higher temperatures (e.g., ~35 MPa, Tenthorey & Cox, 2006). With cohesion, the slip criterion for intergranular flow is assumed to have the form of a Coulomb‐Mohr‐type criterion: $\;\tilde{\tau }={\tilde{S}}_{0}+\tilde{\mu }{\tilde{\sigma }}_{n}$, where ${\tilde{S}}_{0}$ is the cohesion of grain contacts that might be different from the measured macroscopic one (Niemeijer & Spiers, 2007). With this new criterion, the macroscopic friction coefficient can be rewritten as (Niemeijer & Spiers, 2007, their equation (20)):

(A1) $$\mu =\frac{\pi }{\mathrm{zH}}\frac{{\tilde{S}}_{0}}{\sigma_{n}}\frac{\tan\psi }{\cos\psi -\tilde{\mu }\sin\psi }+\frac{\tilde{\mu }+\tan\psi }{1-\tilde{\mu }\tan\psi }.$$

Here, *z* is the grain coordination number, *H* is a geometrical factor (as in A2). Without the first term on the right‐hand side, equation A1 reduces to the original CNS model (cf. equation 1c). Recall that tan*ψ* is defined as

(A2) $$\text{tanψ}=2H\left(\varphi_{c}-\varphi \right),$$

where *φ* falls in the range from *φ*_0_ to *φ*_*c*_. Using typical values for a carbonate gouge (*z* = 6, *H* = 0.567, *φ*_*c*_ = 0.2, and *φ*_0_ = 0.02), *ψ* is typically less than 0.20 such that tan*ψ* ≈ sin*ψ* ≈ *ψ*. Equation A1 can be thus approximated as

(A3) $$\mu \approx \frac{{\tilde{S}}_{0}}{\sigma_{n}}\frac{\sin\psi }{\cos\psi -\tilde{\mu }\sin\psi }+\frac{\tilde{\mu }+\tan\psi }{1-\tilde{\mu }\tan\psi }=\frac{\tilde{\mu }+\left(1+\frac{{\tilde{S}}_{0}}{\sigma_{n}}\right)\tan\psi }{1-\tilde{\mu }\tan\psi }.$$

As given in the main text, the maximum frictional healing is defined as

(A4) $$\max\left(∆\mu_{\mathrm{pk}}\right)=\max\left(\Delta \varphi \right)\frac{\partial \mu }{\partial \varphi }.$$

Taking the partial derivative *∂μ*/*∂φ* from (A3), and substituting the result into (A4), yields

(A5) $$\max\left(∆\mu_{\mathrm{pk}}\right)=2H\left(\varphi_{c}-\varphi_{0}\right)\frac{\left(1+{\tilde{\mu }}^{2}\right)+\frac{{\tilde{S}}_{0}}{\sigma_{n}}}{{\left(1-\tilde{\mu }\tan\psi \right)}^{2}}.$$

This relation gives the limit of frictional healing caused by both compaction and cohesion at grain contacts. Multiplying (A5) by normal stress gives the absolute fault restrengthening (in units of Pa), of which the contribution from cohesion is

(A6) $$C_{\max}=\frac{2H\left(\varphi_{c}-\varphi_{0}\right)}{{\left(1-\tilde{\mu }\tan\psi \right)}^{2}}\;{\tilde{S}}_{0}.$$

Using typical values for a carbonate gouge gives $C_{\max}\approx 0.20{\tilde{S}}_{0}$. Thus, one can generally conclude that the (macroscopic) fault cohesion is smaller than the (microscopic) grain contact cohesion (*C* < $\;{\tilde{S}}_{0}$). This also means that the macroscopic cohesion quoted earlier needs to be multiplied by a porosity factor in order to get the grain contact cohesion.
