# Supplementary material for: Microphysical Model Predictions of Fault Restrengthening Under Room‐Humidity and Hydrothermal Conditions: From Logarithmic to Power‐Law Healing
Source: J Geophys Res Solid Earth. 2020 Apr 3;125(4):e2019JB018567. doi: 10.1029/2019JB018567 (PMC7374943; doi:10.1029/2019JB018567)
Supplement: Supplementary file 1 — Supporting Information S1 [file JGRB-125-e2019JB018567-s001.docx]

Supporting Information for

# Microphysical model predictions of fault restrengthening under room-humidity and hydrothermal conditions: from logarithmic to power-law healing

Jianye Chen^1,2*^, Martijn P.A. van den Ende^1,3^, André R. Niemeijer^1^

Affiliations

*^1^ HPT Laboratory, Department of Earth Sciences, Utrecht University, Utrecht, the Netherlands*

*^2^ Now at Faculty of Civil Engineering and Geosciences, Technical University of Delft, Delft, the Netherlands*

*^3^ Université Côte d’Azur, CNRS, IRD, Observatoire de la Côte d’Azur, Géoazur, 250 rue Albert Einstein, Sophia-Antipolis 06560 Valbonne, France*

** Corresponding author: Jianye Chen E-mail: j.chen3@uu.nl*

**Contents of this file:** Text S1, Figures S1-S5, and Table S1

Text S1 analytically derives the approximation from Eq. (9a) to (9b), which is also numericaly justified in Figure S1. Figure S2 gives the evolution of sample slip rate and its two components during an (interseimic) hold time predicted by the CNS model. Figure S3 presents the sensitivity of the porosity function to stress exponent (*n*-value) of the contact creep law. Figure S4 compares the steady-state friction profiles against log(velocity) for a calcite gouge deformed at hydrothermal and dry conditions. Figure S5 shows the simulation results for a series of SHS tests with different hold times. Figure S6 fits the natural fault healing data reported by Marone et al. (1995) by both a logarithimc and a power law.

Table S1 gives the analytical expressions for equivalent rate-and-state frictional parameters that are derived from our previous work (Chen et al., 2017). Table S2 includes the natural fault healing data reported by Marone et al. (1995).

**Supplement Text S1**: An approximation from Eq. (9a) into Eq. (9b)

As given in Eq. (9a), we have the analytical solution for the evolution of normalized state ($\Psi$) defined as$\Psi\equiv\left( tan\psi/tan\psi_{ss} \right)^{3}$,

$\Psi\left( t_{*} \right)=\frac{1+r{(t_{*}+1)}^{\left( r+1 \right)}}{\left( r+1 \right){(t_{*}+1)}^{r}}$ (S1 or 9a)

Here *t_*_* is a normalized time (*t_*_* = *t*/*t_in_*) and *r* is a (constant) ratio between two characterized times that *r* =$\frac{t_{in}}{t_{c}}=\frac{a\sigma_{n}}{KD_{c}}$. Using typical parameter values from experiments (*a* ~ 0.001 - 0.01, $K$/$\sigma_{n}$~ 5 mm^-1^, *D_c_* ~ 2 - 20 μm), *r* is a small number less than 1 (0.01 - 1).

For long times (*t_*_* >>1), the above relation can be approximated as

$\Psi\left( t_{*} \right)\approx\frac{r}{\left( r+1 \right)}\left( t_{*}+1 \right)\approx\frac{r}{\left( r+1 \right)}t_{*}$. (S2)

For short times (*t_*_* < 1), the above relation can be approximated as

$\Psi\left( t_{*} \right)\approx\frac{1+r\left[ 1+\left( r+1 \right)t_{*} \right]}{\left( r+1 \right)\left( 1+rt_{*} \right)}=1$. (S3)

With much sacrifice of accuracy, Eq. (S2) and (S3) can be combined into Eq (9b):

$\Psi\left( t_{*} \right)\sim\frac{r}{\left( r+1 \right)}t_{*}+1$ (S4 or 9b)

Numerical computation of these two relations (S1 and S4) for different parameter *r* shows that Eq (9b) can accurately capture Eq (9a).


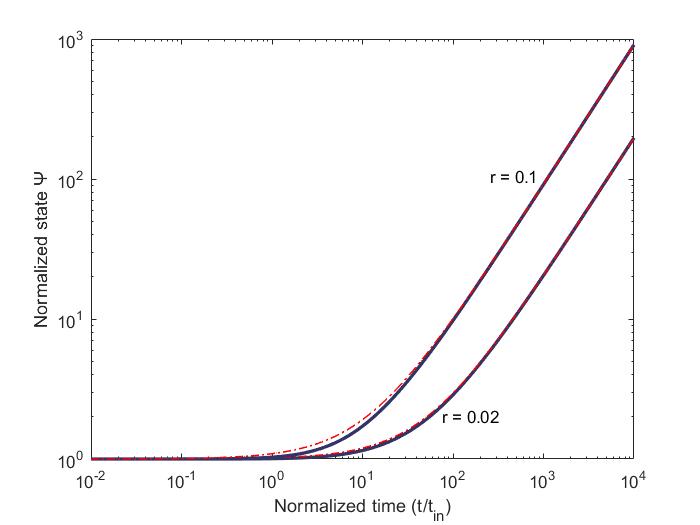


Figure S1. Evolution of the normalized state$(\Psi\equiv\left( tan\psi/tan\psi_{ss} \right)^{3})$ as a function of normalized time ($t_{*}=t/t_{in}$). The solid and dashed lines are the results computed from Eq. 9a and Eq (9b), respectively. The controlling parameter *r* of 0.02 and 0.1 is used.


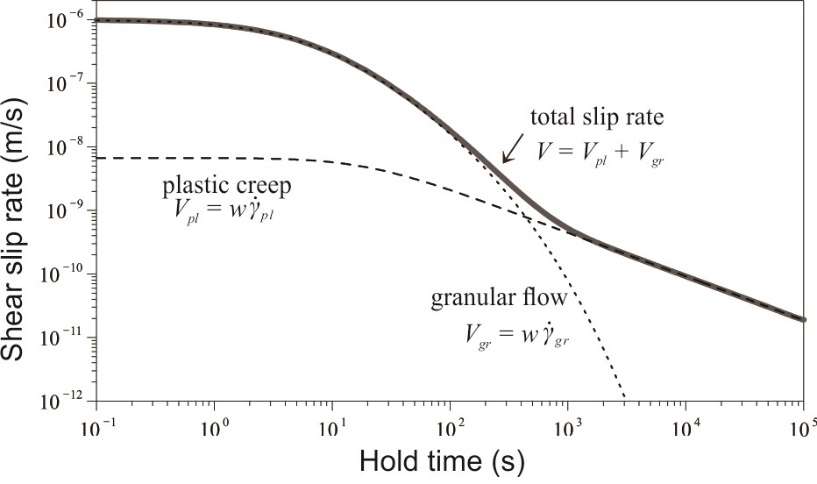


Figure S2. a) Evolution of sample slip rate (*V*, thick line) and its two components (*V_gr_* and *V_pl_*, dashed lines) as a function of hold time. The slip rate at the preceding sliding period is 1 μm/s.


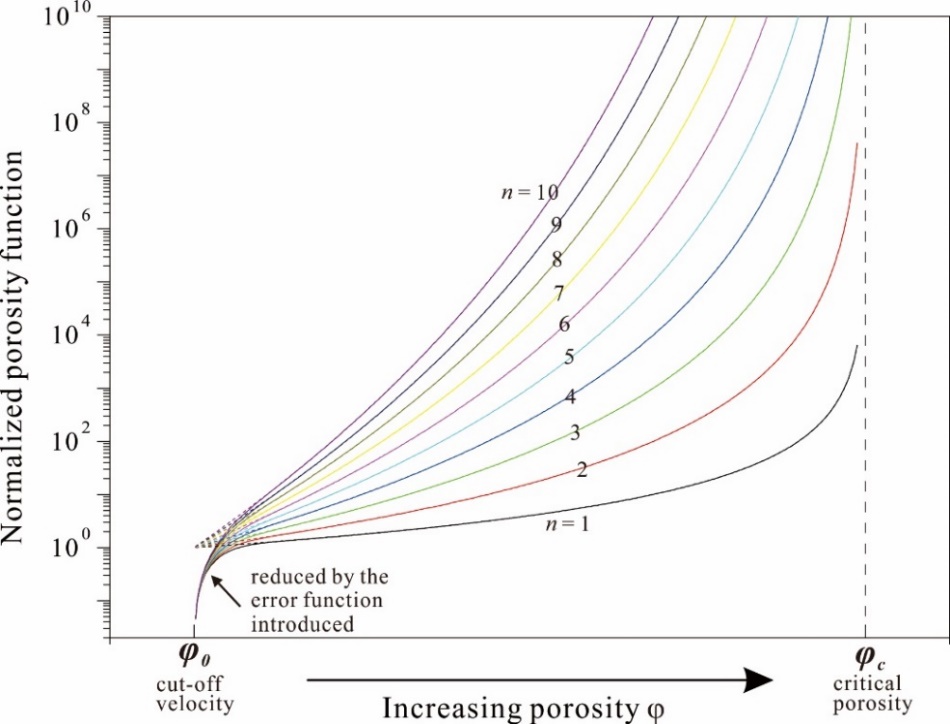


Figure S3. The apparent porosity function that incorporates the stress exponent, plotted for various *n*-values (Eq. 14).


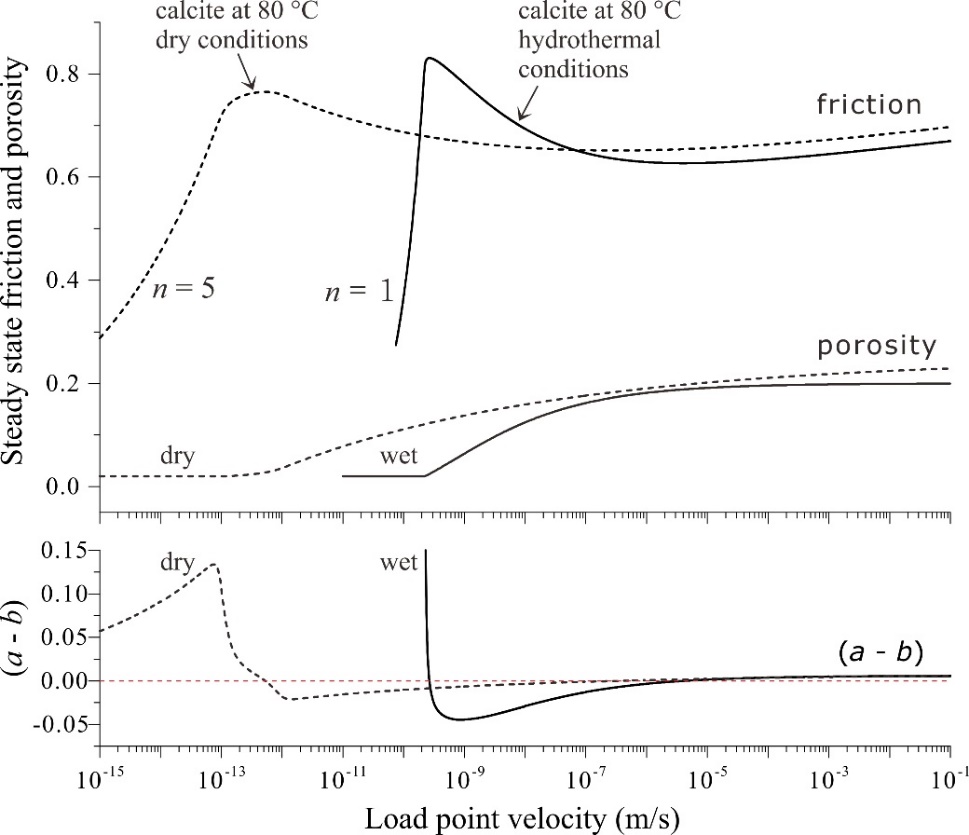


Figure S4. Predicted steady-state friction coefficient and its rate dependence (*a* – *b*), as well as the steady-state porosity, as a function of logarithmic velocity for a calcite fault gouge sheared under hydrothermal and room-humidity conditions. For both conditions, the friction profiles predicted show consecutive transitions from stable to potentially unstable and back to stable slip regimes, and the corresponding (*a* – *b*) values change from positive to negative and back to positive, as load point velocity increases. The porosity profiles show the same trends for both conditions: porosity is at the cutoff level *φ*_0_ in the flow regime, while in the frictional regime, their values increase and asymptotically approach *φ*_c_ as slip rate increases. The main differences between the two cases are that 1) in the flow regime, the strength profile shows different slopes, which depend on the stress exponent of the creep law; 2) the flow-to-friction transition occurs at a lower velocity for the “dry” gouge than the wet one; 3) in the frictional, velocity-weakening regime, the frictional strength is less sensitive to slip rate in the “dry” gouge, giving a minimum (*a* – *b*) of –0.02 versus of –0.05 for the wet gouge; and 4) the “dry” gouge has a higher porosity than the wet one at a given velocity, which is due to the assigned higher critical porosity. Notwithstanding that the rheological properties for the “dry” gouge are poorly constrained, the predictions for the two cases are generally consistent with the common measurements of frictional strength, rate dependence, and porosity of fault gouges from previous experiments (e.g., Chen et al., 2015; Verberne et al., 2013).


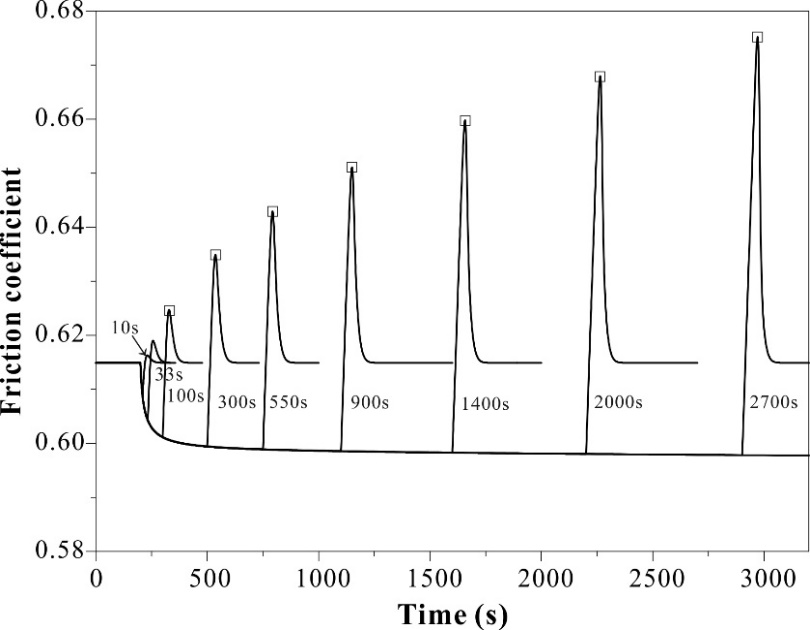


Figure S5. Numerical simulation of a series of SHS tests, showing the evolution of friction coefficient with hold time. The squares give the prediction of peak friction from the analytical expression.


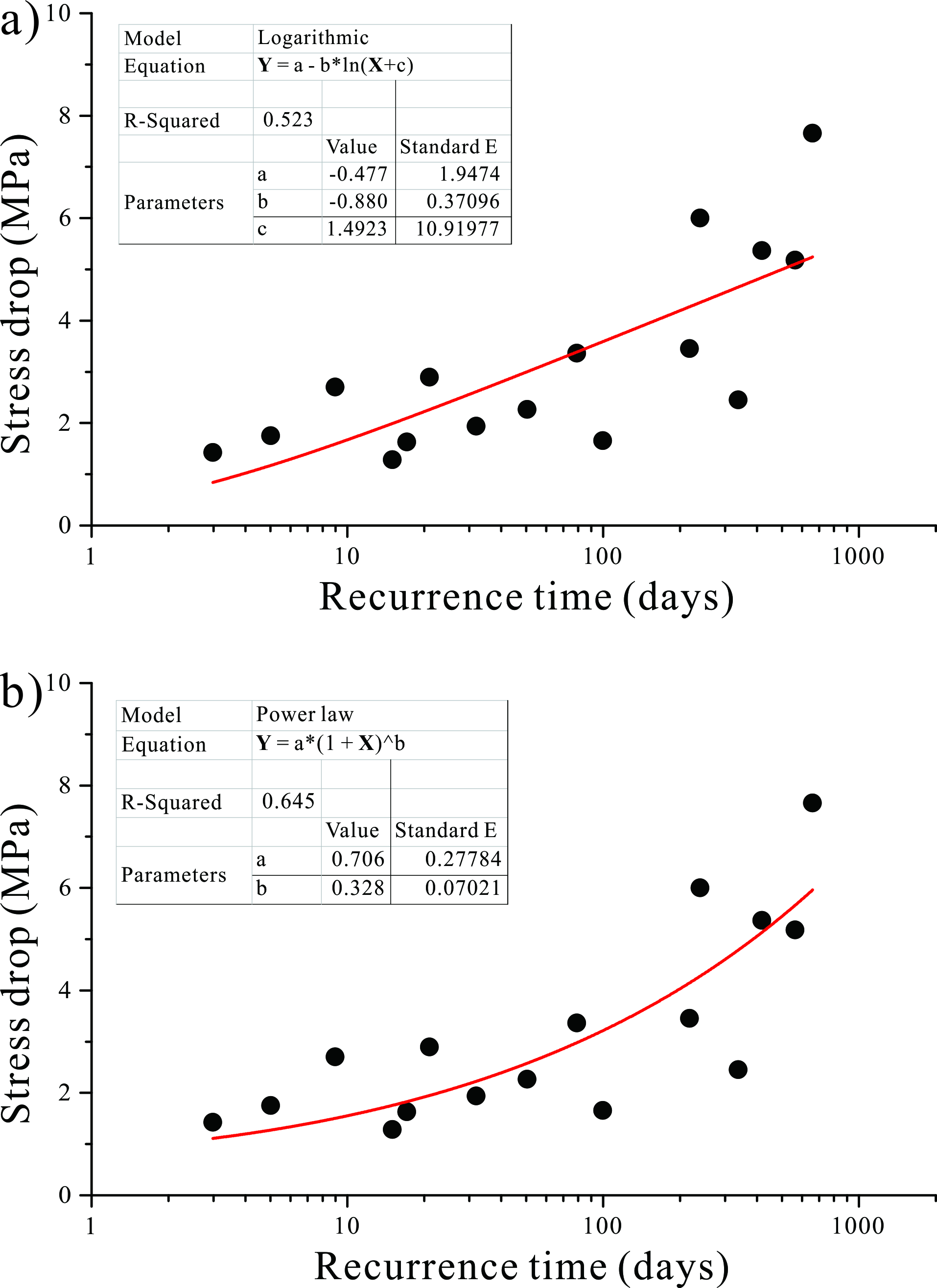


Figure S6. Fitting of the natural fault healing data with a) a logarithmic versus b) power law. The data was extracted from Figure 5a of Marone et al. (1995), with constant rupture velocity (see Table S2). The fitting functions, parameter values and standard errors (95% confidence) are given in the inset tables in a) and b), respectively. In a), the fitting parameters indicate a 2.02 (±0.85) MPa/decade change in stress drop (*b*-value* ln10), consistent with the result given by Marone et al. (1995). In b), the fitting gives a power exponent is 0.328 (±0.07), consistent with the theoretical value of 1/3 predicted by the CNS model.

**Table S1.** **Microphysical Interpretation of Rate-and-State Frictional Parameters**

| Parameters | Explanation | Microphysically-derived expressions |
| --- | --- | --- |
| *μ* | Friction coefficient | $\frac{\tilde{\mu}+\hat{\mu}}{1-\tilde{\mu}\hat{\mu}}$ |
| *a* | Direct effect | $a_{\tilde{\mu}}\frac{1+{\hat{\mu}_{ss}}^{2}}{\left( 1-\tilde{\mu}_{ss}\hat{\mu}_{ss} \right)^{2}}a_{\tilde{\mu}}$ |
| *b* | Evolution effect | $b_{\psi}\frac{1+{\tilde{\mu}_{ss}}^{2}}{\left( 1-\tilde{\mu}_{ss}\hat{\mu}_{ss} \right)^{2}}$ |
| *D_c_* | Characteristic slip distance | $\frac{w}{2H\left( 1+M \right)\left( 1-\varphi_{ss} \right)}$ |

See also Table 1 of Chen et al. (2017). In the present study, the *N*-value in Chen et al. (2017) is set to be 1 and shear deformation is assumed to uniformly distributed over a slip zone of *w* in thickness. Note that $\tilde{\mu}$ is the grain boundary friction coefficient (Eq. 1d) and $\hat{\mu}\equiv tan\psi$, is defined as the friction strength due to dilatation. Furthermore, $a_{\tilde{\mu}}$ and $b_{\psi}$ specify the logarithmic rate dependences of $\tilde{\mu}$ and $\hat{\mu}$, respectively.

**Table S2. Extracted Data from Figure 5a of Marone et al. (1995)**

| Recurrrence time (yr) | Stress drop (MPa) | Recurrrence time (yr) | Stress drop (MPa) |
| --- | --- | --- | --- |
| 2.98466  5.01312  8.97179  14.98708  17.08964  20.99221  31.9179  50.42434 | 1.42273  1.74934  2.70469  1.28391  1.63094  2.89249  1.93714  2.26376 | 79.05333  99.68929  217.95613  239.19685  337.61564  417.44426  563.97553  659.48417 | 3.362  1.65544  3.45182  5.99942  2.45156  5.3666  5.18288  7.65699 |
